# Evaluation of tri-plate rapid on-farm culture system to make therapeutic decisions for mastitis cases in dairy cattle

**DOI:** 10.1371/journal.pone.0353527

**Published:** 2026-07-09

**Authors:** Sajjad Ahmed, Jawaria Ali Khan, Muhammad Avais, Muhammad Imran Rashid, Muhammad Asad Ali

**Affiliations:** 1 Department of Veterinary Medicine, Faculty of Veterinary Science, University of Veterinary and Animal Sciences, Lahore, Pakistan; 2 Department of Clinical Medicine, Faculty of Veterinary Sciences, Lasbela University of Agriculture, Water and Marine Sciences, Uthal, Pakistan; 3 Department of Parasitology, University of Veterinary and Animal Sciences, Lahore, Pakistan; 4 Institute of Microbiology, Faculty of Veterinary Science, University of Veterinary and Animal Sciences, Lahore, Pakistan; PRISM CRO, PAKISTAN

## Abstract

The empirical use of antibiotics for clinical mastitis is a principal driver of antimicrobial resistance in dairy farming. While on-farm culture systems represent a promising strategy for targeted therapy, robust evidence of their efficacy in heterogeneous commercial settings is still needed. We conducted a randomized controlled trial across 16 commercial dairy farms. Cows with clinical mastitis (CM) were allocated to a Positive Control Treatment (PCT) group (n = 57), receiving immediate empirical intramammary (IMM) antibiotics, or a Culture Based Group (CBG) (n = 46), where treatment was directed by a tri-plate on-farm culture system after 24-hour incubation. The cows were considered experimental unit with mixed-effect models within cluster correlation. Data was statistically analyzed using chi square tests, paired t-tests and Kaplan Meier survival analysis via SPSS (version 20.0). The culture-guided protocol enabled a reduction in antibiotic use, eliminating treatment for the 45.6% of CBG cases (no bacterial growth or Gram-negative infections). The clinical cure rates between the groups (CBG 82.6% vs. PCT 75.4%) were not statistically significant (p = 0.28). Similarly, bacteriological cure rates were comparable between (PCT 71.9% vs. CBG 71.7%, p = 0.987). However, the CBG approach revealed significantly lower treatment failure rate (17.3% vs. 24.5%, p < 0.001) with a shorter median time to clinical cure (3 days vs. 7 days, p < 0.001). At the herd level, the strategy was associated with a significant increase in milk yield (+6.94 L/day, p < 0.001) and reduction in somatic cell count (−56.8%, p < 0.001). The tri-plate on-farm culture system is an effective antimicrobial stewardship tool, facilitating a substantial reduction in antibiotic use while accelerating clinical recovery and improving udder health in commercial dairy operations.

## Introduction

Dairy cattle play an important role in agriculture economy in Pakistan and many other South Asian countries, contributing substantially to household food security, employment, and national Gross Domestic Product (GDP) [1]. However, sustainability of dairy industry is particularly challenged by mastitis mainly caused by group of pathogenic bacteria [2]. This infection is an inflammation of mammary gland primarily in cows, resulting decreased milk yield and quality, increased veterinary costs, and early removal of cows [3]. The etiology of this disease is complex, involving a diverse community of bacterial pathogens, including gram-positive organisms such as *Staphylococcus aureus* and streptococci, as well as gram-negative bacteria like *Escherichia coli*. [4]. In line with this, the traditional management of clinical mastitis (CM), usually implicates the instant, and empirical usage of intramammary (IMM) antibiotics, before the causative pathogen is identified [5]. This problematic method has two main downsides; firstly, approximately 30–50% of CM cases are caused by Gram-negative pathogens or due to culture-negative results, for which antibiotic therapy is often ineffective or unnecessary [6]. Second and the most crucial, CM is the main driver of whole antibiotic usage in cow herd. This extensive use of antibiotics is the key factor in the emergence of antimicrobial-resistant (AMR), a profound threat to both veterinary and human medicine [7]. For instance, the AMR has documented dairy in dairy-borne bacteria such as *Listeria monocytogenes* in raw milk [8]. In addition, environmental pathogens such as *E.coli*, and *Klebsiella* spp. are commonly associated with CM, and their effective control requires not only appropriate measures like antibiotic usage but hygiene and bedding management [9]. Moreover, multi-drug resistant *E.coli* has been isolated from environmental sources, underscoring the wider AMR challenge not restricted to CM [10].

To minimize these challenges, selective treatment strategies for the detection and management of mastitis pathogens are gaining acceptance [11,12]. On farm culture (OFC) systems are progressively employed in dairy farms, offering a rapid and cost-effective alternative to laboratory-based diagnostics. Nevertheless, studies demonstrated that OFC can significantly mitigate antibiotics use (40–50%), long-term udder health [13,14], without negatively affecting clinical outcomes, milk production, or udder health. [15,16]. Furthermore, the several studies originates from controlled research herds or specific geographical regions, leaving a gap in understanding of its operational efficacy within heterogeneous farm systems [17,18]. In particular, the tri-plate culture system is the major tool, providing differentiation in major pathogens such as *Staphylococcus aureus*, Streptococci, and Gram-negative bacteria directly on the farm [19]. While the general concept of OFC is supported, the implementation of a precise treatment protocol dictated by the results of this particular tri-plate system constitutes a novel approach [20].

Thus, the current study aimed to assess Tri-plate fast OFC approach as evaluation system for CM. To accomplish this, the randomized trial based study identified and isolated the principle pathogenic bacteria from clinical samples in different dairy farms. Further, implemented a treatment regimen where antibiotic therapy was guided by findings of OFC Tri-plate system.

## Materials and methods

### Study design, randomization and blinding

A randomized controlled field **trial** was carried out on 16 commercial dairy farms in Punjab, Pakistan. Cows diagnosed with CM were randomly allocated at cow level to IMMs. Formal sample size calculation was not performed, owing to routine clinical practices, and absence of prior effect size estimates for the primary outcomes within the local context. The sample size was (≥100 CM cases) was determined on the basis of feasibility constraints to ensure robust analysis. Randomization employed a computer-generated sequence (block 4), stratified by farm [21], with allocation concealed in opaque and sealed envelopes prepared by an independent statistician. The partial blinding was applied: farmers, milkers, laboratory personnel and data analysts were blinded while veterinarians were not blinded due to protocol-specific interventions.

### Case enrolment, and randomization

Cows diagnosed with CM on the basis of aberrations in the milk; clots, color changes, and signs and symptoms of udder and teats (inflammation), such as pain, swelling, and redness. Post diagnosis period, the cows were randomly allocated to one of two treatment groups. The positive control treatment group (PCT) received immediate blanket intramammary antibiotic therapy, representing the traditional empirical practice. While the cultured based group (CBG) received therapeutic choices directed by the results of the OFC tri-plate assay, enabling selective therapy.

#### Sample collection and transportation.

A total of 103 milk samples were collected aseptically from clinically affected quarters. Before collection, the udder was thoroughly cleaned and disinfected by removing any dirt, debris, or bedding material, followed by washing with fresh water and drying with a towel. The teat was then swabbed with alcohol, and after discarding a few strips of milk (22), a 10 ml sample was collected in a pre-labeled, clean falcon-tube. Immediately, 2 mL portion was used to inoculate the on-farm tri-plate culture for CBG. While, the remaining 8 mL was stored at 4°C, and transported to the Animal Health Research Laboratory at Department of Veterinary Medicine, UVAS Lahore, within 4–6 hours for confirmatory analyses.

### On-farm diagnostic procedures

We used the CMT as primary screening tool to support clinical diagnosis of CM. Subsequently, the 2 mL of milk portion was streaked onto the tri-plate, which contained three specialized media compartments: Media-1 for *Staphylococcus aureus* (Mannitol Salt Agar), Media-2 (Blood Agar) for *Streptococcus* species**,** and Media 3 (MacConkey Agar) for Gram-negative bacteria. The plates were incubated at 37°C for 24 hours, and growth was interpreted directly on the farm using standardized guide based colony morphology. Based on culture results, IMM antibiotics were administered following previous guidelines on the basis of tri-plate findings at 24 hours. Moreover, quarters with no bacterial growth, or gram-negative received supportive care, while quarters gram positive and mixed growth administered narrow spectrum and broad spectrum antibiotics respectively (Table 1).

**Table 1:** Treatment protocol for clinical mastitis based on on-farm tri-plate culture results in the CBG.

On-farm culture result	Therapeutic decision	Intramammary antibiotic	Spectrum	Dosage and duration
Gram-positive growth	Targeted antibiotic therapy	Cloxacillin (500 mg)	Narrow	1 tube per quarter, every 24 hours for 3 days
Gram-negative growth	Supportive care only	None	–	–
Mixed growth	Broad-spectrum antibiotic therapy	Amoxicillin (200 mg)/ Clavulanic Acid (50 mg)	Broad	1 tube per quarter, every 24 hours for 3 days
No growth	Supportive care only	None	–	

#### Bacteriological analysis.

The SCC was determined using fluorescent flow cytometry. Quarters were considered unhealthy with a thresholds value (>200,000 cells/mL). The milk samples were cultured onto three standard culture media; Blood Agar (general identification), MacConkey Agar (gram-negative), and Mannitol Salt Agar (selective medium, *Staphylococcus aureus)*. The plates were incubated at 37°C for 24–48 hours. The aerobic culture procedures for cold milk samples collected on farms adhered to the established guidelines set by the National Mastitis Council [22] and as described by [17].

### Bacterial confirmation

Bacterial isolates were identified based on colony morphology through Gram staining, and series of biochemical tests including catalase, coagulase, oxidase, CAMP testing, and esculin hydrolysis.

### Ethics statement

The research protocol was reviewed and approved by the Institutional guidelines and ethical review committee of the University of Veterinary and Animal Sciences (UVAS) Lahore, Pakistan (DR/459).

### Statistical analysis

Data was statistically analyzed using R (version 4.3.2) and SPSS (version 20.0). The cow was considered one independent observation unit for both primary and secondary clinical outcomes. The continuous variables including milk yield, SCC, and days to cure, were expressed as mean ± standard deviation, following confirmation of normality with the Shapiro-Wilk test. The paired samples t-tests were used for pre-post treatment comparisons. Similarly, the categorical variables including cure rates, culture findings, and treatment failure were expressed as frequencies (%) and analyzed using Pearson’s chi-square tests on raw cow level count(s). The clinical status was evaluated merely on days 3, 5, and 7 post-enrollment, thus day-to-cure are interval censored. Time-to-event data (days to clinical cure) were assessed with Kaplan-Meier survival analysis and the log-rank test. Multiple linear regression models were constructed to identify predictors of post-treatment milk yield and SCC, with diagnostic checks for homoscedasticity and inter-correlation. The data was considered statistically significant with p- value < 0.05.

## Results

### Demographic characteristics

Current study enrolled 103 cows affected with clinical mastitis from 16 commercial dairy farms of Punjab Pakistan. Of these, 57 cows were allocated to PCT group, while 46 cows were assigned to CBG. The PCT was provided with immediate intramammary antibiotic therapy, and CBG in which therapeutic choices were based on the result of a rapid on-farm tri-plate culture assay. The tri-plate rapid on farm culture was successfully implemented and utilized to direct treatment in CBG. There were significant (p= < 0.001) difference in initial culture findings of both groups. The culture results from CBG revealed that 45.7% cows were diagnosed with gram positive infection, followed by 6.5% gram negative, 8.7% mixed infection, and 39.1% with no bacterial growth while PCT did not undergo initial culture (Table 2). This diagnostic classification served as the foundation for the cultured based treatment strategy in the CBG group. Based infection site in the herds, our analysis revealed the highest infection rate in single quarter (64.1%), followed by double quarter (25.2%), and triple (10.7%). Similarly, the infection distributed relatively among four udder quarters such as Left Rear (LR, 17.5%), Right Front (RF, 16.5%), Left Front (LF, 16.5%), and Right Rear (RR, 13.6%), and at the same time multiple quarters (RF, LF, RR, 13.6%)

**Table 2:** Baseline Demographic and Clinical Characteristics of Cows with Clinical Mastitis, by Treatment Group.

Characteristics	Total (n = 103)	PCT Group (n = 57)	CBG Group (n = 46)	p-value
Clinical Presentation				
Number of Infected Quarters, n (%)	N (%)	N (%)	N (%)	0.451
1	66 (64.1)	38 (66.7)	28 (60.9)	
2	26 (25.2)	12 (21.1)	14 (30.4)	
3	11 (10.7)	7 (12.3)	4 (8.7)	
Quarter Position	N (%)	–	–	--
Single Quarter Infections				
Right Front (RF)	17 (16.5)			
Left Rear (LR)	18 (17.5)			
Right Rear (RR)	14 (13.6)			
Left Front (LF)	17 (16.5)			<0.001
Right Rear (RR)	14 (13.6)			
Multiple Quarter Infections				
RF, RR, LF	14 (13.6)				
Culture Findings	N (%)	N (%)	N (%)	
Gram-Positive	21 (20.4)	0 (0.0)	21 (45.7)	
Gram-Negative	3 (2.9)	0 (0.0)	3 (6.5)	
Mixed	4 (3.9)	0 (0.0)	4 (8.7)	
No Growth	18 (17.5)	0 (0.0)	18 (39.1)	
NA (PCT)	57 (55.3)	57 (100.0)	0 (0.0)

Note: Data in rows such as number of infected Quarters and culture findings are presented at the cow level (n = 103 cows). Similarly, information shown for single quarter infections and multiple quarter infections are at the quarter level only (n = 151 quarters) to describe baseline infection distribution and were not the independent observations used in outcome analyses. PCT = positive control treatment, CBG = culture-based group, NA = not applicable.

### Primary and secondary treatment outcomes

In this study, the primary and secondary outcomes between the groups were compared in context of different variables. The study revealed insignificant (p = 0.281) differences in the primary outcomes of initial clinical cure in PCT group (75.4%) and CBG (82.6%,), while bacteriological cure rates observed no statistical significance (p = 0.987) in PCT (41/57, 71.9%%), and CBG (33/46, 71.7%, p = 0.987, Table 3). For secondary outcomes CBG method significantly (p < 0.001) reduced (CBG 8/46, 17.3% vs. PCT 14/57, 24.5%), and significantly (p < 0.001) reduced mean days of clinical cure (CBG, 3.04 ± 0.29 days vs. PCT, 6.50 ± 0.94 days). In contrast, post-treatment new infection rate was (CBG 9/46, 19.6% vs. PCT 15/57, 26.3%, p = 0.987) and mean treatment duration (CBG 3.10 ± 1.62 days vs. PCT 3.08 ± 0.49 days) were observed statistically insignificant (p = 0.891).

**Table 3:** Comparison of Primary and Secondary Treatment Outcomes between PCT and CBG Groups.

Outcome(s)	Total (n = 103)	PCT Group (n = 57)	CBG Group (n = 46)	p-value
Primary Outcome				
Clinical Cure, n (%)	75/103 (72.8)	43/57 (75.4%)	38/46 (82.6%)	0.281
Bacteriological Cure, n (%)	74 (71.8%)	41/57 (71.9%)	33/46 (71.7%)	0.987
Secondary Outcome				
Treatment Failure, n (%)	22/103 (21.4%)	14/57 (24.5%)	8/46 (17.3%)	0.001
New Infection Post-Treatment (%)	24 (23.3%)	15/57 (26.3%)	9 (19.6%)	0.987
Days to Clinical Cure, Mean ± SD	4.96 ± 1.878	6.50 ± 0.94	3.04 ± 0.29	0.001
Treatment Duration (days), Mean ± SD	3.08 ± 0.78	3.078 ± 0.49	3.10 ± 1.62	0.891

**Fig 1:**
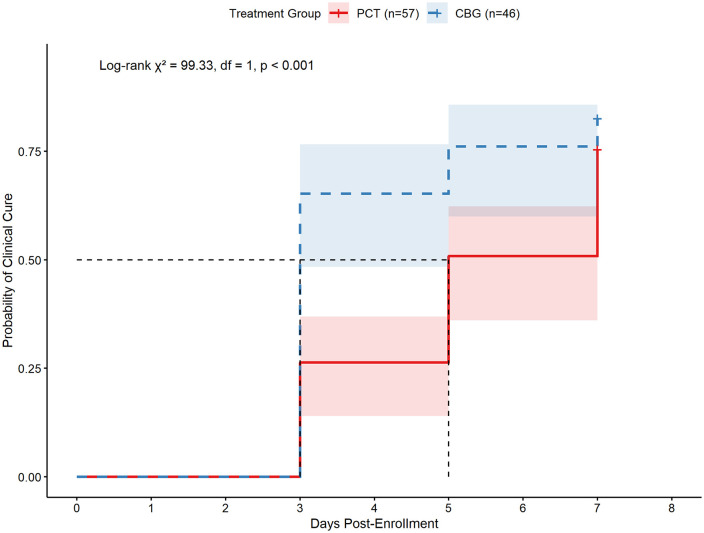
Kaplan-Meier survival curves of cumulative clinical cure over time. The figure illustrates the cumulative probability clinically cure over time in the PCT group (n = 57) and in the CBG, (n = 46). Clinical status was measured at Day 3, 5 and 7 post-enrollment only, therefore days-to-cure are interval-censored and the curves step only at those time points. Median time to clinical cure was 3 days (CBG) vs. 7 days (PCT). A significant difference between groups was observed using the log-rank test (χ² = 99.33, df = 1, p= < 0.001). The shaded areas represent 95% confidence intervals.

According to Kaplan-Meier survival analysis as presented in (Fig 1) revealed that CBG treatment program lead rapid clinical cure with a median recovery time of 3 days compared to PCT group (7 days). The analysis shown that most of the infected cows (75%) on CBG program cured by day 3, while half of the herd on PCT program cure at day 7, which indicates that half of the cows from the this group had experienced treatment failure. According to Mantel Cox test, CBG program significantly (Log-rank test: χ² = 99.33, df = 1, p = < 0.001) effect days to clinical cure compared to PCT group. These findings revealed a significant evidence that CBG practices in farms was higher in results compared to PCT protocols, inhibiting the negative outcome under examination.

### Herd-level response to therapy and predictors of post-treatment milk yield and SCC

**Fig 2:**
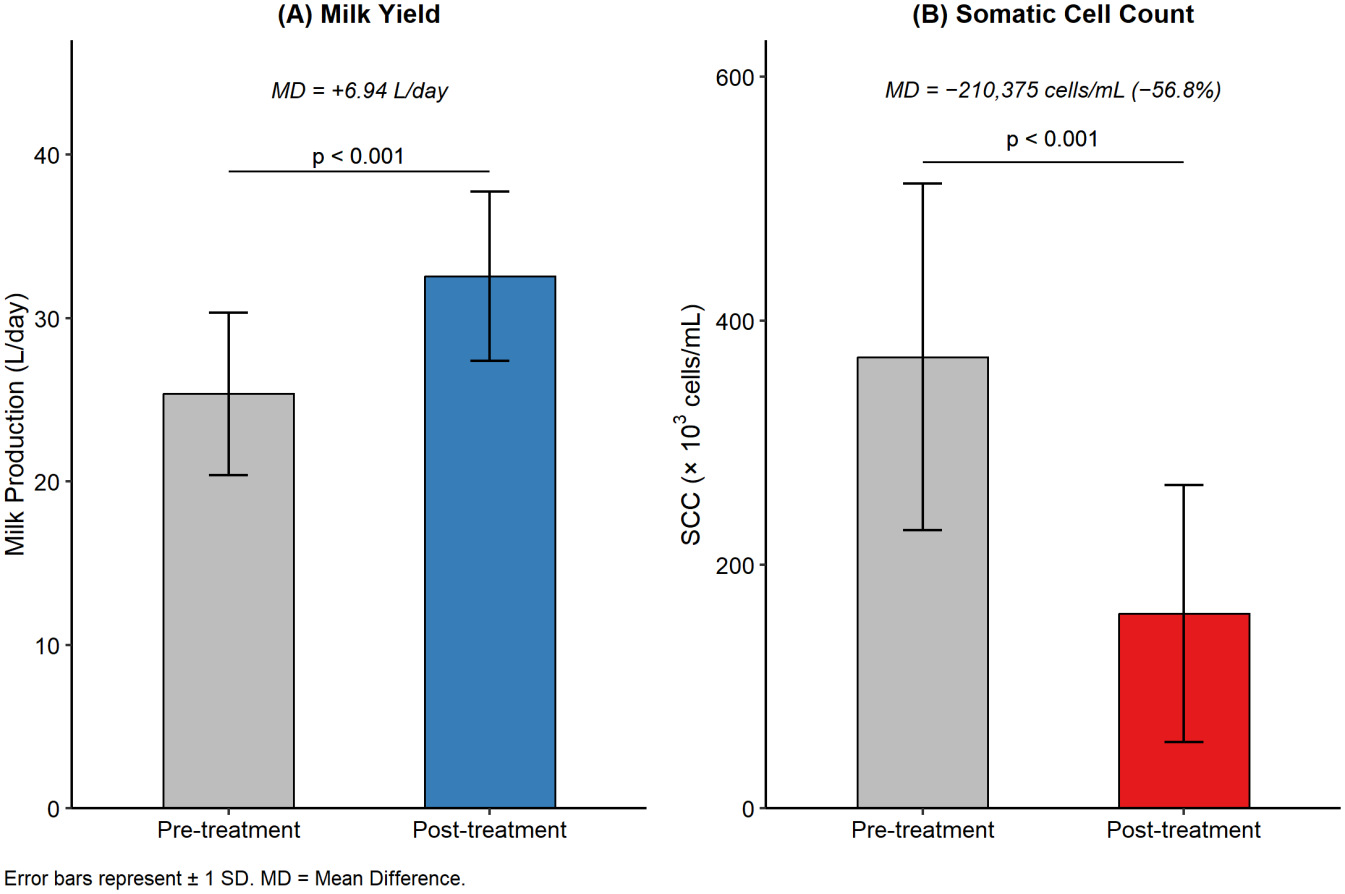
Pre- and post-treatment changes in milk yield and SCC (n = 103). (A) Mean daily milk production increased significantly from 25.36 ± 4.97 L/day pre-treatment to 32.57 ± 5.18 L/day post-treatment with MD = +6.94 L/day, p < 0.001). (B) Mean SCC decreased significantly from 370,234 ± 142,063 cells/mL pre-treatment to 159,859 ± 105,334 cells/mL post-treatment (MD = −210,375 cells/mL, −56.8%, p=< 0.001). Error bars represent ± 1 standard deviation (SD).

Current study shown significant health improvement after treatment in udder parameters such as milk production improved significantly (25.36 ± 4.97 L/day-32.57 ± 5.18 L/day, p= < 0.001) with mean difference (MD) of +6.94 L/day (Table 4 and Fig 2). Simultaneously, the mean SCC significantly (p < 0.001) decreased from 370234.1 ± 142062.8 cells/mL to 159859.2 ± 105334.1cells/mL (MD −336.0 cells/mL). The post treatment outcome was analyzed using multiple linear regression model. According to the model, baseline milk production, higher infected quarters (β = 0.591, p < 0.001) and significantly affected the post-treatment production. While the model revealed 33.9% (F, 3, 99 = 16.94, p= < 0.001), of the variance in post-treatment milk production. Similarly, the baseline SCC significantly demonstrated strong positive predictor (β = 0.960, p < 0.001), while the elucidated 92% of the variance in post-treatment SCC (F (3, 99) = 377.19, p= < 0.001).

**Table 4:** Pre- and Post-Treatment Changes in Milk Yield and Somatic Cell Count (Paired Analysis, n = 103).

Variable	Pre-treatment (Mean ± SD)	Post-treatment (Mean ± SD)	Mean Difference (95% CI)	t-statistic (df)	p-value
Milk Production (L/day)	25.36 ± 4.97	32.57 ± 5.18	+6.94 (4.38 to 5.50)	19.43 (102)	<0.001
Somatic Cell Count (cells/mL)	370234.1 ± 142062.8	159859.2 ± 105334.1	−210,374.91 (−56.8%)	14.3(102)	<0.001

### Prevalence and etiology of mastitis on farm level

The present study identified various types of pathogens (gram positive, gram negative) and prevalence (0.1% to 14.0%) of mastitis. The gram positive *Staphylococcus aureus* and *Streptococcus* spp. and gram negative *E.coli* were identified the most prevalent pathogens across the farms (S1 Table). Similarly, wide range of other gram positive isolates such as *Staph. epidermidis, Strep. Agalactiae, C. bovis, S. pneumoniae, S. pyogenes,* and gram negative pathogens *(Klebsiella* spp., *Pseudomonas* spp) were also reported.

### Management and environmental factors

Current study revealed sand based stall systems along with sand or sawdust bedding as farm management (S2 Table). While the farm hygiene was evaluated on scale of 1–5 and ranged from 2–5, demonstrating variation in whole farm hygiene. The herds in all farms comprised of mainly Holstein-Friesian (HF) cows from different origins.

## Discussion

### Antimicrobial stewardship impact

The empirical use of antibiotics in livestock contributes to AMR. In dairy farming, CM represents a primary reason for antimicrobial administration [23,24]. Our randomized controlled trial demonstrates that a culture-based treatment strategy for CM, guided by a rapid on-farm tri-plate system, significantly reduces antibiotic use while maintaining clinical cure rates.

The most direct impact of the CBG protocol was the substantial reduction of antibiotic in 45.6% all CBG cases to supportive care without antibiotics (39.1% no bacterial growth and 6.5% Gram-negative growth). This strategic withholding corresponds with antimicrobial stewardship (AMS) principle. For instance, self-limiting Gram-negative pathogens (*E.coli)*, and the host immune responses often perform bacterial clearance without antibiotics [25]. Administering antibiotics in such cases offers limited clinical benefit while exerting unnecessary selection pressure for resistance. Our results provide field-based validation confirms that this principle, previously reported in controlled studies [18,26], can be operationalized effectively on commercial farms, align with international AMS guidelines [27].

### Clinical outcomes

A critical finding was that this reduction in antibiotic use was achieved without compromising treatment efficacy. We found an insignificant difference between the CBG and PCT groups in the primary outcomes of clinical cure (82.6% vs. 75.4%, p = 0.28) and bacteriological cure (71.8% vs. 71.9%, p > 0.987). These comparable findings are consistent with previous research reporting maintained clinical outcomes while reducing antibiotic use by 40–50% through culture-guided decisions [18,28,29]. It robustly confirms that blanket therapy for all CM cases is unnecessary.

Notably, CBG protocol demonstrated significant clinical advantages: lower treatment failure rate (PCT 24.5% vs. CBG 17.3%, p < 0.001) and a faster median time to clinical cure (3 days vs. 7 days). The targeted therapy based on rapid 24-hour diagnosis facilitates more rapidly, appropriate therapy against the causative pathogen without delay, rather than relying on a broad-spectrum empirical approach that may be ineffective against the specific causative agent. Our findings are in agreement with previous studies [28,30].

### Farm level variability, pathogen profile, and herd level effects

Moreover, OFC system confirmed the prevalence of different Gram-positive (45.7%, *Staphylococcus aureus*, *Streptococcus* spp. etc.), followed by mixed infection (8.7%), and Gram negative (6.5%) infections, highly consistent with previous studies [31,32]. This variability highlights the need the inconsistency in prevalence and pathogen profile farm specific knowledge to guide treatment decisions. The management data that includes the introduction of sand-based stalls and the variations in the hygienic scores (2–5) revealed that the environmental factors were likely factors contributing to the risk of mastitis. Studies claimed that poor hygiene and high bacterial count in stalls are significant environmental risk contributor for CM [33,34].

At herd level, significant improvement was noted in udder health after treatment. A significant reduction was noticed in the bulk tank SCC 159,859.2 ± 105,334.1 post-treatment is crucial findings of our study. These values reduced significantly from a common industrial threshold of 200,000 cells/mL, which indicate good udder health. Consequently, this alleviation reduced the level of intra-mammary inflammation and mitigated bacterial load within the herd. Parallel to udder health, our study revealed significant increase in milk yield (32.56 ± 5.18, + 6.94 L/day). As the inflammation reduces (reflect SCC reduction), secretary and production capability in the mammary gland restored, followed by milk synthesis and good quality. The findings of our study strongly consistent with previous studies [35,36]. Several studies argued the inverse relationship between milk yield and SCC severe CM, where higher SCC level constantly reduced milk production [18,37,38].

### Implementation, limitation and consideration(s)

The successful implementation of the tri-plate OFC system was fundamental to our outcomes. This system provided (Mannitol Salt Agar, Blood Agar, and MacConkey Agar), a pragmatic and cost-effective diagnostic solution, enabling pathogen-based therapeutic decisions within a clinically relevant timeframe. Our findings supported previous work [39], demonstrating that using OFC guidelines to selective treatment decreases antibiotics usage to considerable amount (40–50%), irrespective of cure rate(s) or milk yield. Nevertheless, OFC systems fluctuate; requires independent and rigorous evaluation under field conditions to confirm its efficacy and practicality for farmers and veterinarians.

Future studies with larger, multi-regional cohorts would strengthen these findings. Furthermore, the operational efficacy of the on-farm culture system depends on proper training in aseptic sampling and plate interpretation. Future research should also investigate the long-term economic impact of this approach, balancing the initial diagnostic costs against savings from reduced antibiotic use, lower milk discard, and improved herd productivity.

### Limitations

There are several limitations in this study. Formal sample size calculation was not performed, because there were no prior effect size estimates in the local context, so our claims of equivalence between groups should be viewed as “no significant difference” rather than formal non-inferiority. Secondly, clinical cure was determined only on Days 3, 5, and 7 after enrollment, with days-to-cure interval-censored; the actual time to cure could have been on any day during the interval between the clinical examinations. Thirdly, the treating veterinarians were not blinded to group assignment because of protocol-specific interventions, which may create detection bias. Fourth, our sample was collected from 16 farms in one region of Pakistan, and our findings may not be applicable to other production systems. Fifth, the on-farm culture system demands training of personnel to undertake the aseptic sample and to interpret the plates, and this can reduce the repeatability of the system in less controlled environments.

### Conclusions

This study demonstrates that a rapid on-farm tri-plate culture system can effectively guide selective treatment decisions for clinical mastitis in commercial dairy herds. The culture-based approach achieved clinical and bacteriological cure rates comparable to empirical blanket therapy, while significantly reducing antibiotic use, shortening time to clinical recovery, and lowering treatment failure rates. These findings support the integration of on-farm culture systems as a practical AMS strategy to improve udder health and promote responsible antimicrobial use in dairy production.

## Supporting information

S1 Table Farm-wise Distribution of Pathogen and Prevalence of Clinical Mastitis. (DOCX)

S2 Table Management and Environmental Factors Associated with Mastitis Prevalence. (DOCX)

S1 File Data. (SPV)
