# Supplementary material for: Evaluation of tri-plate rapid on-farm culture system to make therapeutic decisions for mastitis cases in dairy cattle
Source: PLoS One. 2026 Jul 9;21(7):e0353527. doi: 10.1371/journal.pone.0353527 (PMC13349151; doi:10.1371/journal.pone.0353527)
Supplement: S1 Table — (DOCX) [file pone.0353527.s001.docx]

S1 Table. Farm-wise distribution of pathogen and prevalence of clinical mastitis.

| Farm | Herd | Positive Cases | Prevalence (%) | *Streptococcus (%)* | *Staph. aureus (%)* | *E. coli (%)* | Mixed Infections (%) | Other Isolates *(%)* |
| --- | --- | --- | --- | --- | --- | --- | --- | --- |
| 1 | 250 | 7 | 1.3 | 66 | — | 33 | 33 | — |
| 2 | 190 | 5 | 13.3 | 66 | — | 33 | — | — |
| 3 | 100 | 5 | 5.5 | 33 | 33 | 16 | 33 | *Staph. epidermidis (50)* |
| 4 | 110 | 5 | 3.8 | — | 40 | — | — | *—* |
| 5 | 110 | 6 | 1.6 | 40 | 40 | — | 20 | *Staph. sp. (40)* |
| 6 | 100 | 7 | 8.0 | — | 100 | — | 60 | *Klebsiella (50), Staph. epi (50), Strep. agalactiae (25)* |
| 7 | 130 | 4 | 1.1 | — | — | — | — | *Klebsiella (33), Pseudomonas (16)* |
| 8 | 56 | 4 | 0.9 | — | — | — | 16 | *—* |
| 9 | 36 | 4 | 6.3 | — | 40 | — | 20 | *Staph. epidermidis (80)* |
| 10 | 450 | 6 | 7.0 | — | 42 | — | — | *—* |
| 11 | 270 | 4 | 0.7 | 25 | 25 | 25 | 25 | *—* |
| 12 | 60 | 4 | 2.1 | — | 33 | 50 | 16 | *—* |
| 13 | 150 | 8 | 10.8 | — | 23 | — | — | *C. bovis (7), S. epi (7), S. pneumoniae (7), S. pyogenes (7)* |
| 14 | 150 | 11 | 6.9 | 33 | 88 | 33 | 100 | *S. epidermidis (100)* |
| 15 | 157 | 14 | 14.0 | 15 | 23 | 15 | 30 | *S. epi (7), Strep. agalactiae (15)* |
| 16 | 557 | 11 | 0.1 | 16 | — | 16 | 16 | — |
