# Supplementary material for: Evaluation of tri-plate rapid on-farm culture system to make therapeutic decisions for mastitis cases in dairy cattle
Source: PLoS One. 2026 Jul 9;21(7):e0353527. doi: 10.1371/journal.pone.0353527 (PMC13349151; doi:10.1371/journal.pone.0353527)
Supplement: S2 Table — (DOCX) [file pone.0353527.s002.docx]

S2 Table. Management and environmental factors associated with mastitis prevalence.

| Farm | Hygiene Score (1–5) | Stall Type | Bedding Type | Breed | Location |
| --- | --- | --- | --- | --- | --- |
| 1 | 4 | Sand | Sawdust | HF (US) | Gujranwala |
| 2 | 3 | Sand | Sand | HF (AUS) | Gujranwala |
| 3 | 5 | Sand | Sawdust | Cross-bred HF (AUS) | Sheikhupura |
| 4 | 3 | Sawdust | Sand | HF (AUS) | Sheikhupura |
| 5 | 4 | Sand | Sand | HF (AUS) | Narowal |
| 6 | 2 | Sand | Sand | HF (AUS) | Sahiwal |
| 7 | 4 | Sand | Sawdust | HF (AUS) | Lahore |
| 8 | 4 | Sand | Sand | HF (US) | Nankana Sahib |
| 9 | 3 | Sand | Sand | HF (Dutch) | Lahore |
| 10 | 3 | Sawdust | Sawdust | HF (AUS) | Kasur |
| 11 | 4 | Sand | Sand | HF (US) | Kasur |
| 12 | 4 | Sand | Sand | HF (AUS) | Sahiwal |
| 13 | 4 | Sawdust | Sawdust | HF (AUS) | Kasur |
| 14 | 4 | Sand | Sand | HF (AUS) | Faisalabad |
| 15 | 3 | Sand | Sand | HF (US) | Sahiwal |
| 16 | 3 | Sand | Sand | HF (US, AUS) | Kasur |
