# Supplementary material for: Evaluation of tri-plate rapid on-farm culture system to make therapeutic decisions for mastitis cases in dairy cattle
Source: PLoS One. 2026 Jul 9;21(7):e0353527. doi: 10.1371/journal.pone.0353527 (PMC13349151; doi:10.1371/journal.pone.0353527)
Supplement: Attachment — Submitted filename: PONE-D-25-65785_review ONP.pdf [file pone.0353527.s004.pdf]

PLOS One

Evaluation of Tri-plate Rapid On-Farm Culture System to Make Therapeutic Decisions for Mastitis Cases in Dairy Cattle

--Manuscript Draft--

**Manuscript Number:**	PONE-D-25-65785
**Article Type:**	Research Article
**Full Title:**	Evaluation of Tri-plate Rapid On-Farm Culture System to Make Therapeutic Decisions for Mastitis Cases in Dairy Cattle
**Short Title:**	Tri-Plate Culture Guides Mastitis Treatment
**Corresponding Author:**	Sajjad Ahmad, PhD<br>University of Veterinary and Animal Sciences<br>Lahore, Punjab PAKISTAN
**Keywords:**	assessment; clinical mastitis; treatment; on farm culture; Tri-plate assay
**Abstract:**	The empirical use of antibiotics for clinical mastitis is a principal driver of antimicrobial resistance in dairy farming. While on-farm culture systems represent a promising strategy for targeted therapy, robust evidence of their efficacy in heterogeneous commercial settings is still needed. We conducted a randomized controlled trial across 16 commercial dairy farms. Cows with clinical mastitis were allocated to a Positive Control Treatment (PCT) group (n=57), receiving immediate empirical intramammary (IMM) antibiotics, or a Culture Based Group (CBG) (n=46), where treatment was directed by a tri-plate on-farm culture system after 24-hour incubation. Data was statistically analyzed using R (version 4.3.2) and SPSS (version 20.0). The culture-guided protocol enabled a drastic reduction in antibiotic use, eliminating treatment for the 45.6% of CBG cases with no bacterial growth or Gram-negative infections. Clinical and bacteriological cure rates were statistically non-inferior between the CBG and PCT groups. However, the CBG approach resulted in a significantly lower treatment failure rate (19.35% vs. 80.65%, p<0.001) and a shorter median time to clinical cure (3 days vs. 7 days, p=0.001). At the herd level, the implementation of this strategy was associated with a significant increase in milk yield (+6.94 L/day, p<0.001) and a concurrent reduction in somatic cell count (-56.8%, p<0.001). This study provides compelling evidence that a tri-plate on-farm culture system is an effective antimicrobial stewardship tool. Its implementation facilitates a substantial reduction in antibiotic use while simultaneously accelerating clinical recovery and improving overall udder health in commercial dairy operations.
**Order of Authors:**	Sajjad Ahmad, PhD
	Jawaria Ali, PhD
	Muhammad Avais, PhD
	Imran Rashid, PhD
	Muhammad Asad Ali, PhD
**Additional Information:**	
**Question**	**Response**
**Financial Disclosure**<br><br>Enter a financial disclosure statement that describes the sources of funding for the work included in this submission. Review the submission guidelines for detailed requirements. View published research articles from *PLOS ONE* for specific examples.	Yes

Powered by Editorial Manager® and ProduXion Manager® from Aries Systems Corporation

This statement is required for submission and **will appear in the published article** if the submission is accepted. Please make sure it is accurate.<br><br>**Funded studies**<br>Enter a statement with the following details:<br>• Initials of the authors who received each award<br>• Grant numbers awarded to each author<br>• The full name of each funder<br>• URL of each funder website<br>• Did the sponsors or funders play any role in the study design, data collection and analysis, decision to publish, or preparation of the manuscript?<br><br>Did you receive funding for this work?	
Please add funding details.<br>as follow-up to "**Financial Disclosure**<br><br>Enter a financial disclosure statement that describes the sources of funding for the work included in this submission. Review the submission guidelines for detailed requirements. View published research articles from *PLOS ONE* for specific examples.<br><br>This statement is required for submission and **will appear in the published article** if the submission is accepted. Please make sure it is accurate.<br><br>**Funded studies**<br>Enter a statement with the following details:<br>• Initials of the authors who received each award<br>• Grant numbers awarded to each author<br>• The full name of each funder<br>• URL of each funder website<br>• Did the sponsors or funders play any role in the study design, data collection and analysis, decision to publish, or preparation of the manuscript?	This research was supported by the Punjab Agricultural Research Board (PARB) under the Competitive Grants System (Project ID No. 21-39).

Did you receive funding for this work?"	
Please select the country of your main research funder (please select carefully as in some cases this is used in fee calculation).<br>as follow-up to "**Financial Disclosure**<br><br>Enter a financial disclosure statement that describes the sources of funding for the work included in this submission. Review the submission guidelines for detailed requirements. View published research articles from *PLOS ONE* for specific examples.<br><br>This statement is required for submission and **will appear in the published article** if the submission is accepted. Please make sure it is accurate.<br><br>**Funded studies**<br>Enter a statement with the following details:<br>• Initials of the authors who received each award<br>• Grant numbers awarded to each author<br>• The full name of each funder<br>• URL of each funder website<br>• Did the sponsors or funders play any role in the study design, data collection and analysis, decision to publish, or preparation of the manuscript?<br><br>Did you receive funding for this work?"	PAKISTAN - PK
**Competing Interests**<br><br>Use the instructions below to enter a competing interest statement for this submission. On behalf of all authors, disclose any competing interests that could be perceived to bias this work—acknowledging all financial support and any other relevant financial or non-financial competing interests.<br><br>This statement is required for submission and **will appear in the published article** if	The authors have declared that no competing interests exist.

the submission is accepted. Please make sure it is accurate and that any funding sources listed in your Funding Information later in the submission form are also declared in your Financial Disclosure statement.<br><br>View published research articles from [*PLOS ONE*] for specific examples.<br><br>**NO authors have competing interests**<br><br>Enter: *The authors have declared that no competing interests exist.*<br><br>**Authors with competing interests**<br><br>Enter competing interest details beginning with this statement:<br><br>*I have read the journal's policy and the authors of this manuscript have the following competing interests: [insert competing interests here]*<br><br>* typeset	
**Ethics Statement**<br><br>Enter an ethics statement for this submission. This statement is required if the study involved:<br><br>• Human participants<br>• Human specimens or tissue<br>• Vertebrate animals or cephalopods<br>• Vertebrate embryos or tissues<br>• Field research<br><br>Write "N/A" if the submission does not require an ethics statement.<br><br>General guidance is provided below. Consult the submission guidelines for detailed instructions. **Make sure that all information entered here is included in the Methods section of the manuscript.**	The research protocol was reviewed and approved by the institutional guidelines and ethical review committee of the University of Veterinary and Animal Sciences (UVAS), Lahore, Pakistan (DR/459).

**Format for specific study types**<br><br>**Human Subject Research (involving human participants and/or tissue)**<br>• Give the name of the institutional review board or ethics committee that approved the study<br>• Include the approval number and/or a statement indicating approval of this research<br>• Indicate the form of consent obtained (written/oral) or the reason that consent was not obtained (e.g. the data were analyzed anonymously)<br><br>**Animal Research (involving vertebrate animals, embryos or tissues)**<br>• Provide the name of the Institutional Animal Care and Use Committee (IACUC) or other relevant ethics board that reviewed the study protocol, and indicate whether they approved this research or granted a formal waiver of ethical approval<br>• Include an approval number if one was obtained<br>• If the study involved *non-human primates*, add *additional details* about animal welfare and steps taken to ameliorate suffering<br>• If anesthesia, euthanasia, or any kind of animal sacrifice is part of the study, include briefly which substances and/or methods were applied<br><br>**Field Research**<br><br>Include the following details if this study involves the collection of plant, animal, or other materials from a natural setting:<br>• Field permit number<br>• Name of the institution or relevant body that granted permission	
**Data Availability**<br><br>Authors are required to make all data underlying the findings described fully available, without restriction, and from the time of publication. PLOS allows rare exceptions to address legal and ethical concerns. See the PLOS Data Policy and FAQ for detailed information.	Yes - all data are fully available without restriction

A Data Availability Statement describing where the data can be found is required at submission. Your answers to this question constitute the Data Availability Statement and **will be published in the article**, if accepted.<br><br>**Important:** Stating 'data available on request from the author' is not sufficient. If your data are only available upon request, select 'No' for the first question and explain your exceptional situation in the text box.<br><br>Do the authors confirm that all data underlying the findings described in their manuscript are fully available without restriction?	
**Describe where the data may be found in full sentences. If you are copying our sample text, replace any instances of XXX with the appropriate details.**<br><br>• If the data are **held or will be held in a public repository**, include URLs, accession numbers or DOIs. If this information will only be available after acceptance, indicate this by ticking the box below. For example: *All XXX files are available from the XXX database (accession number(s) XXX, XXX.).*<br>• If the data are all contained **within the manuscript and/or Supporting Information files**, enter the following: *All relevant data are within the manuscript and its Supporting Information files.*<br>• If neither of these applies but you are able to provide **details of access elsewhere**, with or without limitations, please do so. For example:<br><br>*Data cannot be shared publicly because of [XXX]. Data are available from the XXX Institutional Data Access / Ethics Committee (contact via XXX) for researchers who meet the criteria for access to confidential data.*<br><br>*The data underlying the results presented in the study are available from (include the name of the third party*	All relevant data are within the manuscript and its Supporting Information files

and contact information or URL).<br>• This text is appropriate if the data are owned by a third party and authors do not have permission to share the data.<br><br>* typeset	
Additional data availability information:	

Powered by Editorial Manager® and ProduXion Manager® from Aries Systems Corporation

Main docx Click here to access/download;Manuscript;Main docx.docx

Evaluation of Tri-plate Rapid On-Farm Culture System to Make Therapeutic Decisions for Mastitis Cases in Dairy Cattle

Sajjad Ahmed[1], Jawaria Ali[1], Muhammad Avais[1], Imran Rashid[2,], Muhammad Asad Ali [3]

[1]Department of Veterinary Medicine. Faculty of Veterinary Sciences. University of Veterinary and Animal Sciences Lahore (54200) Pakistan

[2]Department of Parasitology, University of Veterinary and Animal Sciences Lahore (54200) Pakistan

[3]Department of Microbiology, Faculty of Veterinary Science. University of Veterinary and Animal Sciences Lahore (54200) Pakistan

Corresponding author: ahmad.sajjadbu@gmail.com

Abstract

The empirical use of antibiotics for clinical mastitis is a principal driver of antimicrobial resistance in dairy farming. While on-farm culture systems represent a promising strategy for targeted therapy, robust evidence of their efficacy in heterogeneous commercial settings is still needed. We conducted a randomized controlled trial across 16 commercial dairy farms. Cows with clinical mastitis were allocated to a Positive Control Treatment (PCT) group (n=57), receiving immediate empirical intramammary (IMM) antibiotics, or a Culture Based Group (CBG) (n=46), where treatment was directed by a tri-plate on-farm culture system after 24-hour incubation. Data was statistically analyzed using R (version 4.3.2) and SPSS (version 20.0). The culture-guided protocol enabled a drastic reduction in antibiotic use, eliminating treatment for the 45.6% of CBG cases with no bacterial growth or Gram-negative infections. Clinical and bacteriological cure rates were statistically non-inferior between the CBG and PCT groups. However, the CBG approach resulted in a significantly lower treatment failure rate (19.35% vs. 80.65%, p<0.001) and a shorter median time to clinical cure (3 days vs. 7 days, p=0.001). At the herd level, the implementation of this strategy was associated with a significant increase in milk yield (+6.94 L/day, p<0.001) and a concurrent reduction in somatic cell count (-56.8%, p<0.001).This study provides compelling evidence that a tri-plate on-farm culture system is an effective antimicrobial stewardship tool. Its implementation facilitates a substantial reduction in

antibiotic use while simultaneously accelerating clinical recovery and improving overall udder health in commercial dairy operations.

Keywords: Assessment; clinical mastitis; treatment; on farm culture; Tri-plate assay

Introduction

Dairy Cattles play an important role in agriculture economy in Pakistan and many other south Asian countries, contributing substantially to household food security, employment, and national Gross Domestic Product (GDP) [1]. However, sustainability of dairy industry is particularly challenged by mastitis mainly caused by group of pathogenic bacteria [2]. This infection is an inflammation of mammary gland primarily in cows, resulting decreased milk yield and quality, increased veterinary costs, and early removal of cows [3]. The etiology of this disease is complex, involving a diverse community of bacterial pathogens, including gram-positive organisms such as *Staphylococcus aureus* and streptococci, as well as gram-negative bacteria like *Escherichia coli*. [4]. In line with this, the traditional management of clinical mastitis (CM), usually implicates the instant, and empirical usage of intramammary (IMM) antibiotics, before the causative pathogen is identified [5]. This problematic method has two main downsides; firstly, approximately 30-50% of CM cases are caused by Gram-negative pathogens or due to culture-negative results, for which antibiotic therapy is often ineffective or unnecessary [6]. Second and the most crucial, CM is the main driver of whole antibiotic usage in cow herd. This extensive use of antibiotics is the key factor in the emergence of antimicrobial-resistant (AMR), a profound threat to both veterinary and human medicine [7]. To minimize this problem, selective treatment strategies for detection and management of mastitis pathogens are gaining acceptance [8-10]. On farm culture (OFC) systems are progressively employed in dairy farms, offering a rapid and cost-effective and rapid alternative to laboratory-based diagnostics. Nevertheless, studies demonstrated that OFC can significantly mitigate antibiotics use (40-50%), long-term udder health [11, 12], without negatively affecting clinical outcomes, milk production, or udder health. [13-15]. Furthermore, the several studies originates from controlled research herds or specific geographical regions, leaving a gap in understanding of its operational efficacy within heterogeneous farm systems [16, 17]. In particular, the tri-plate culture system is the major tool, providing differentiation in major pathogens such as *Staphylococcus aureus*, Streptococci, and Gram-negative bacteria directly on the farm [18]. While the general concept of

OFC is supported, the implementation of a precise treatment protocol dictated by the results of this particular tri-plate system constitutes a novel approach [19].

Thus, the current study aimed to assess Tri-plate fast OFC approach as evaluation system For CM. Tom accomplish this, this randomized trial based study identified and isolated the principle pathogenic bacteria from clinical samples in different dairy farms. Further, implemented a treatment regimen where antibiotic therapy was guided by findings of OFC Tri-plate system.

Materials and methods

Study design

A randomized controlled field trial was carried out on 16 commercial dairy farms in Punjab, Pakistan. The herd characteristics such as size, housing systems, mean milk yield levels, California Mastitis Test (CMT)**,** and somatic cell count (SCC), were documented. Owing to routine clinical practice, and absence of prior effect size estimates for the primary outcomes within the local context, formal sample size calculation was not conducted. The sample size was determined by practical constraints, with the aim of enrolling of a minimum of 100 CM cases, to ensure for analysis.

Case enrolment, and randomization

Cows diagnosed with CM on the basis of aberrations in the milk; clots, color changes, and signs and symptoms of udder and teats (inflammation), such as pain, swelling, and redness. Post diagnosis period, the quarters were randomly allocated to one of two treatment groups. The positive control treatment group (PCT) received immediate blanket intramammary antibiotic therapy, representing the traditional empirical practice. While the cultured based group (CBG) received therapeutic choices directed by the results of the OFC tri-plate assay, enabling selective therapy.

Sample collection and Transportation

A total of 103 milk samples were collected aseptically from fro clinically effected quarters. Before collection, the udder was thoroughly cleaned and disinfected by removing any dirt, debris, or bedding material, followed by washing with fresh water and drying with a towel. The teat was then swabbed with alcohol, and after discarding a few strips of milk [20], a 10 ml sample was collected in a pre-labeled, clean falcon-tube. Immediately, 2 mL portion was used to inoculate the on-farm tri-plate culture for CBG. While, the remaining 8 mL was stored at 4°C, and transported to the Animal Health Research Laboratory at Department of Veterinary Medicine, UVAS Lahore, within 4-6 hours for confirmatory analyses.

On-Farm Diagnostic Procedures

We used the California CMT as primary screening tool to support clinical diagnosis of CM. Subsequently, the 2 mL of milk portion was streaked onto the tri-plate, which contained three specialized media compartments: Media-1 for *Staphylococcus aureus* (Mannitol Salt Agar), Media-2 (Blood Agar) for *Streptococcus* species**,** and Media 3 (MacConkey Agar) for Gram-negative bacteria. The plates were incubated at 37°C for 24 hours, and growth was interpreted directly on the farm using standardized guide based colony morphology. Based on culture results, IMM antibiotics were administered following previous guidelines on the basis of tri-plate findings at 24 hours. Moreover, quarters with no bacterial growth, or gram-negative received supportive care, while quarters gram positive and mixed growth administered narrow spectrum and broad spectrum antibiotics respectively, as detailed in Table 1.

Table 1. Treatment protocol for clinical mastitis based on on-farm tri-plate culture results in the CBG.

On-farm culture result	Therapeutic decision	Intramammary antibiotic	Spectrum	Dosage and duration
Gram-positive growth	Targeted antibiotic therapy	Cloxacillin (500 mg)	Narrow	1 tube per quarter, every 24 hours for 3 days
Gram-negative growth	Supportive care only	None	–	–
Mixed growth	Broad-spectrum antibiotic therapy	Amoxicillin (200 mg) / Clavulanic Acid (50 mg)	Broad	1 tube per quarter, every 24 hours for 3 days
No growth	Supportive care only	None	–	

Bacteriological Analysis

The SCC was determined using fluorescent flow cytometry. Quarters were considered unhealthy with a thresholds value (>200,000 cells/mL). The milk samples were cultured onto three standard culture media; Blood Agar (general identification), MacConkey Agar (gram-negative), and Mannitol Salt Agar (selective medium, *Staphylococcus aureus)*. The plates were incubated at 37°C for 24-48 hours. The aerobic culture procedures for cold milk samples collected on farms adhered to the established guidelines set by the National Mastitis Council (NMC, 1999) and as described by [17].

Bacterial Confirmation

Bacterial isolates were identified based on colony morphology through Gram staining, and series of biochemical tests including catalase, coagulase, oxidase, CAMP testing, and esculin hydrolysis.

Ethics statement

The research protocol was reviewed and approved by the Institutional guidelines and ethical review committee of the University of Veterinary and Animal Sciences (UVAS) Lahore, Pakistan (DR/459).

Statistical analysis

Data was statistically analyzed using R (version 4.3.2) and SPSS (version 20.0). The continuous variables including milk yield, SCC, and days to cure, were expressed as mean ± standard deviation, following confirmation of normality with the Shapiro-Wilk test. The paired samples t-tests were used for treatment duration before and after treatment. Similarly, the categorical variables such as quarter position, numbers, cure rates, culture findings, and treatment failure were expressed as frequencies (%) and analyzed using Chi-square tests. Time-to-event data (days to clinical cure) were assessed with Kaplan-Meier survival analysis and the log-rank test. Multiple linear regression models were constructed to identify predictors of post-treatment milk yield and SCC, with diagnostic checks for homoscedasticity and inter-correlation. The data was considered statistically significant with p- value < 0.05.

Results

Demographic Characteristics

Current study enrolled 103 cows affected with clinical mastitis in 151 quarters from 16 commercial dairy farms of Punjab Pakistan. Of these, 57 cows with 83 (n=103), with effected quarters were allocated to PCT group, while 46 cows with 68 effected were assigned to CBG. The PCT was provided with immediate intramammary antibiotic therapy, and CBG in which therapeutic choices were based on the result of a rapid on-farm tri-plate culture assay. The tri-

plate rapid on farm culture was successfully implemented and utilized to direct treatment in CBG. There were significant ($p<0.001$) difference in initial culture findings of both groups. The culture results from CBG revealed that 45.7% cows were diagnosed with gram positive infection, followed by 6.5% gram negative, 8.7% mixed infection, and 39.1% with no bacterial growth while PCT did not undergo initial culture, as mentioned in Table 2. This diagnostic classification served as the foundation for the cultured based treatment strategy in the CBG group. Based infection site in the herds, our analysis revealed the highest infection rate in single quarter (64.1%), followed by double quarter (25.2%), and triple (10.7%). Similarly, the infection distributed relatively among four udder quarters such as Left Rear (LR, 17.5%), Right Front (RF, 16.5%), Left Front (LF, 16.5%), and Right Rear (RR, 13.6%), and at the same time multiple quarters (RF, LF, RR, 13.6%)

Table 2. Baseline Demographic and Clinical Characteristics of Cows with Clinical Mastitis, by Treatment Group

Characteristics	Total (n=103)	PCT Group (n=57)	CBG Group (n=46)	p-value
Clinical Presentation				
Number of Infected Quarters, n (%)	N (%)	N (%)	N (%)	0.451
1	66 (64.1)	38 (66.7)	28 (60.9)	
2	26 (25.2)	12 (21.1)	14 (30.4)	
3	11 (10.7)	7 (12.3)	4 (8.7)	
Quarter Position	N (%)	-	-	--
Single Quarter Infections				
Right Front (RF)	17 (16.5)			

Left Rear (LR)	18 (17.5)			
Right Rear (RR)	14 (13.6)			
Left Front (LF)	17 (16.5)			
Right Rear (RR)	14 (13.6)			
Multiple Quarter Infections				
RF, RR, LF	14 (13.6)			
Culture Findings	N (%)	N (%)	N (%)	
Gram-Positive	21 (20.4)	0 (0.0)	21 (45.7)	
Gram-Negative	3 (2.9)	0 (0.0)	3 (6.5)	
Mixed	4 (3.9)	0 (0.0)	4 (8.7)	<0.001
No Growth	18 (17.5)	0 (0.0)	18 (39.1)	
NA (PCT)	57 (55.3)	57 (100.0)	0 (0.0)	

Primary and Secondary Treatment Outcomes

In this study, the primary and secondary outcomes between the groups were compared in context of different variables. The study revealed insignificant (p >0.05) differences in the primary outcomes of initial clinical cure in PCT group (57.33%) and CBG (42.67 %,), while bacteriological cure rates also observed no statistical significance (p>0.577) in PCT (55.41%), and CBG (44.59%, Table 3). However, in the secondary outcomes CBG method significantly reduced (19.35%, p<0.001) treatment failure compared to blanket therapy of PCT group (80.65%). Similarly, CBG observed significant (p=0.001) reduction in the days of clinical cure (3.04± 0.29 days), compared PCT group (6.50 ± 0.94 days). In contrast, new infection rate after treatment (p>0.285) and average treatment duration were observed statistically insignificant (p>0.89).

According to Kaplan-Meier survival analysis as presented in (Figure 1) revealed that CBG treatment program lead rapid clinical cure with median recovery time of 3 days compared to PCT group (7 days). The analysis shown that shown that most of the infected cows (75%) on CBG program cured by day 3, while half of the herd on PCT program cure at day 7, which indicates that half of the cows from the this group had experienced treatment failure. According to Mantel Cox test, CBG program significantly (Log-rank test: $\chi^2 = 99.33$, df=1, p = 0.001) effect days to clinical cure compared to PCT group. These findings revealed a significant evidence that CBG practices in farms was higher in results compared to PCT protocols, inhibiting the negative outcome under examination.

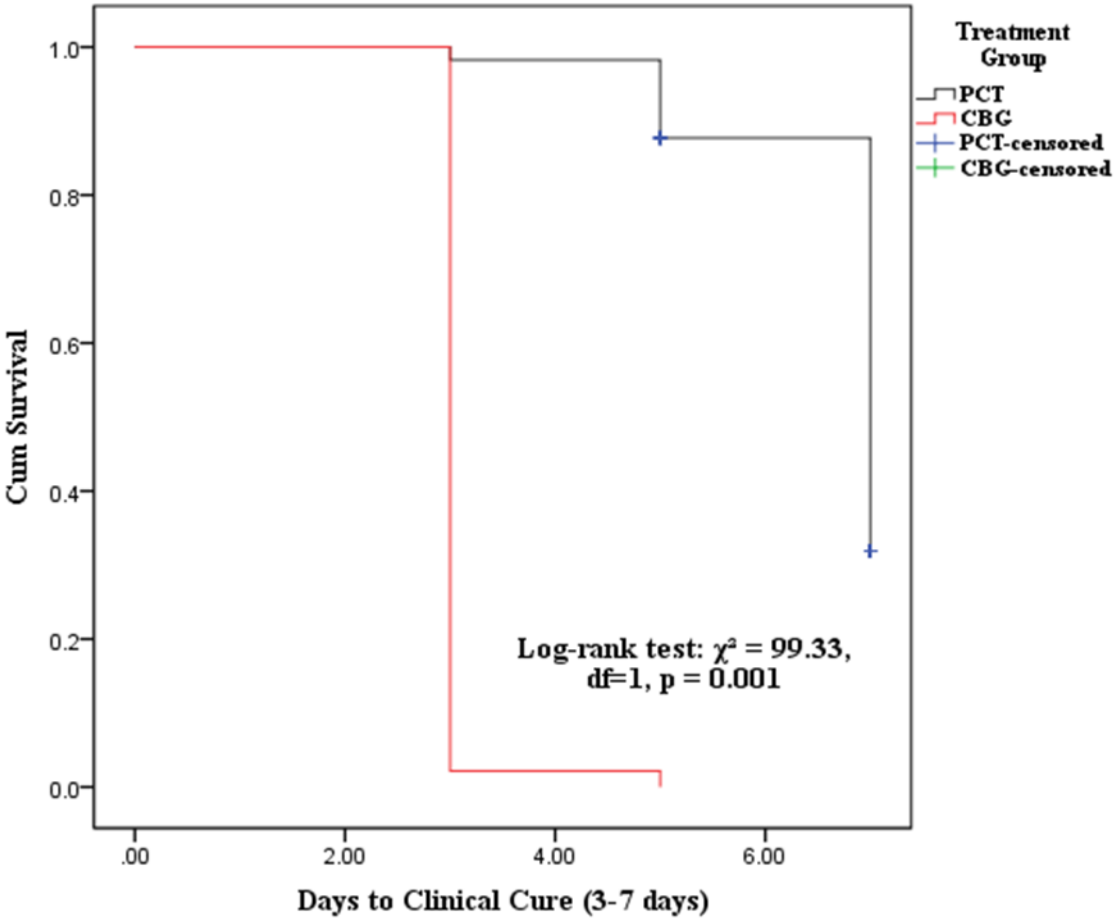

Figure 1: Kaplan-Meier analysis demonstrating probability of a clinical cure at time interval (3- 7

days), between the two treatment groups. The median period to clinical cure was 3 days for CBG and 7 days for PCT. The Mantel Cox test, evaluated group differences (Log-rank test: $\chi^2 = 99.33$, df=1, p = 0.001) affect days to clinical cure.

Table 3. Comparison of Primary and Secondary Treatment Outcomes between PCT and CBG Groups

Outcome(s)	Total (n=103)	PCT Group (n=57)	CBG Group (n=46)	p-value
Primary Outcome				
Clinical Cure, n (%)	75 (72.8)	43 (57.33%)	32 (42.67%)	0.329
Bacteriological Cure, n (%)	74 (71.8)	41(55.41%)	33 (44.59%)	0.577
**Secondary Outcome**				
Treatment Failure, n (%)	31 (30.1)	25 (80.65%)	6 (19.35%)	0.001
New Infection Post-Treatment, n (%)	24 (23.3)	15(62.50%)	9 (37.50%)	0.285
Days to Clinical Cure, Mean ± SD	4.96±1.878	6.50± 0.94	3.04± 0.29	0.001
Treatment Duration (days), Mean ± SD	3.08±0.78	3.078±0.49	3.10±1.62	0.89

Herd-Level Response to Therapy and Predictors of Post-Treatment Milk Yield and SCC

Current study shown significant health improvement after treatment in udder parameters such as milk production improved significantly (25.36 ± 4.97 L/day-32.57 ± 5.18 L/day, p= <0.001) with mean difference (MD) of +6.94 L/day (Table 4 & Fig. 2). Simultaneously, the mean SCC significantly (p=<0.001) decreased from 370234.1 ± 142062.8 cells/mL to 159859.2 ± 105334.1cells/mL (MD -336.0 cells/mL). The post treatment outcome was analyzed using

multiple linear regression model. According to the model, baseline milk production, higher infected quarters ($\beta = 0.591$, $p < 0.001$) and significantly affected the post-treatment production. While the model revealed 33.9% (F, 3, 99 = 16.94, $p < 0.001$), of the variance in post-treatment milk production. Similarly, the baseline SCC significantly demonstrated strong positive predictor ($\beta = 0.960$, $p < 0.001$), while the elucidated 92% of the variance in post-treatment SCC (F (3, 99) = 377.19, $p < 0.001$).

Table 4. Pre- and Post-Treatment Changes in Milk Yield and Somatic Cell Count (Paired Analysis, n=103)

Variable	Pre-treatment (Mean ± SD)	Post-treatment (Mean ± SD)	Mean Difference (95% CI)	t-statistic (df)	p-value
Milk Production (L/day)	25.36 ± 4.97	32.57 ± 5.18	+6.94 (4.38 to 5.50)	19.43 (102)	<0.001
Somatic Cell Count (cells/mL)	370234.1 ± 142062.8	159859.2 ± 105334.1	-210,374.91 (-56.8%)	14.3(102)	<0.001

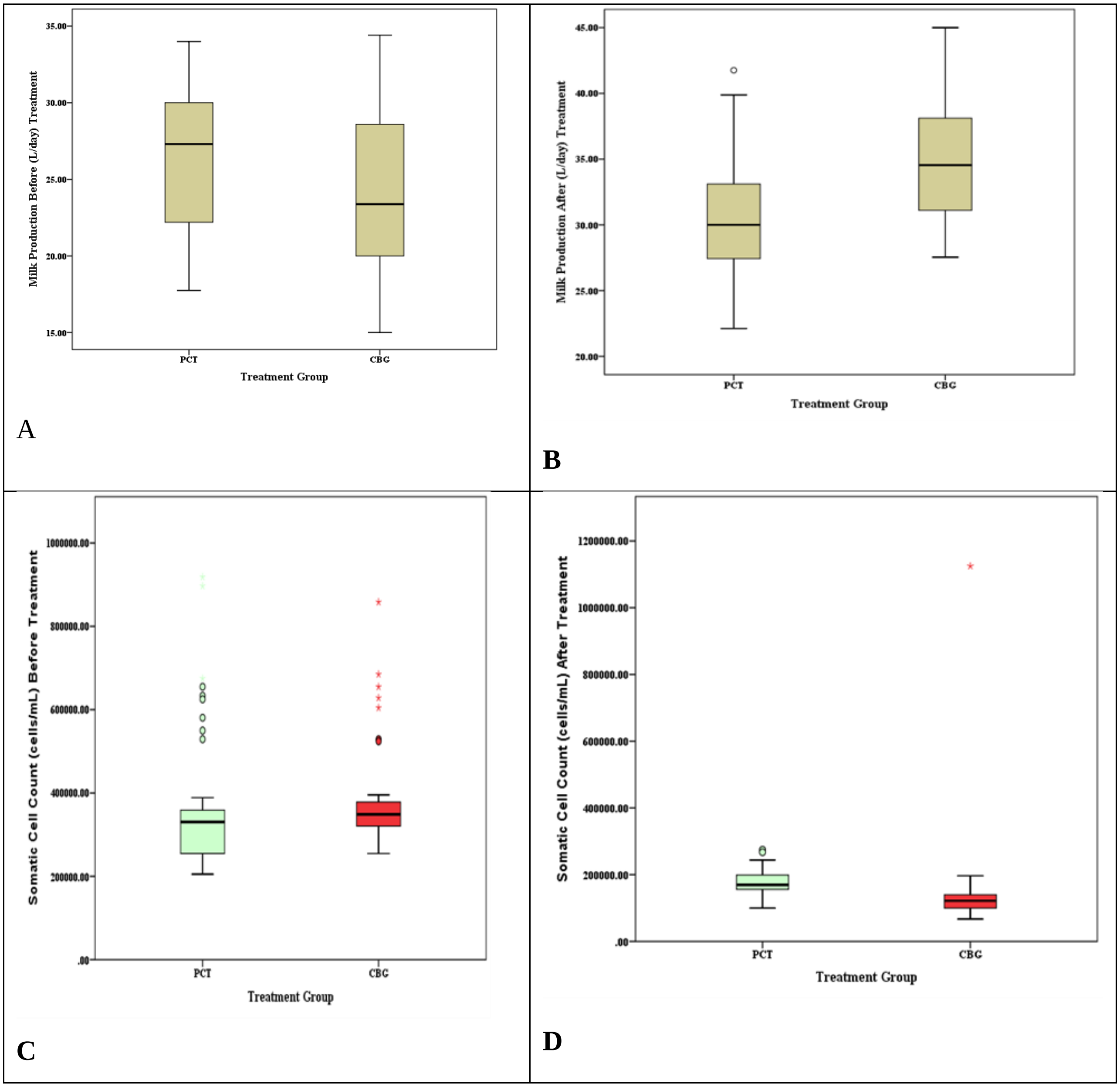

Figure 2: Demonstrates pre- and post-treatment variations in Milk Yield and SCC. The milk production significantly improved ($p < 0.001$) with mean difference (MD) of +6.94 L/day, while the mean of SCC significantly reduced ($p<0.001$) with MD of -210,374.91 cells/ mL (-56.8%)

Prevalence and Etiology of Mastitis on Farm Level

The present study identified various types of pathogens (gram positive, gram negative) and prevalence (0.1% to 14.0%) of mastitis. The gram positive *Staphylococcus aureus* and *Streptococcus* spp. and gram negative *E.coli* were identified the most prevalent pathogens across the farms (Table 5). Similarly, wide range of other gram positive isolates such as *Staph. epidermidis, Strep. Agalactiae, C. bovis, S. pneumoniae, S. pyogenes,* and gram negative pathogens *(* *Klebsiella* spp., *Pseudomonas* spp) were also reported.

Table 5. Farm-wise Distribution of Pathogen and Prevalence Clinical Mastitis

Farm	Herd	Positive Cases	Prevalence (%)	*Streptococcus (%)*	*Staph. aureus (%)*	*E. coli (%)*	Mixed Infections (%)	Other Isolates *(%)*
1	250	7	1.3	66	—	33	33	—
2	190	5	13.3	66	—	33	—	—
3	100	5	5.5	33	33	16	33	*Staph. epidermidis (50)*
4	110	5	3.8	—	40	—	—	—
5	110	6	1.6	40	40	—	20	*Staph. sp. (40)*
6	100	7	8.0	—	100	—	60	*Klebsiella (50), Staph. epi (50), Strep. agalactiae (25)*

7	130	4	1.1	—	—	—	—	*Klebsiella (33), Pseudomonas (16)*
8	56	4	0.9	—	—	—	16	—
9	36	4	6.3	—	40	—	20	*Staph. epidermidis (80)*
10	450	6	7.0	—	42	—	—	—
11	270	4	0.7	25	25	25	25	—
12	60	4	2.1	—	33	50	16	—
13	150	8	10.8	—	23	—	—	*C. bovis (7), S. epi (7), S. pneumoniae (7), S. pyogenes (7)*
14	150	11	6.9	33	88	33	100	*S. epidermidis (100)*
15	157	14	14.0	15	23	15	30	*S. epi (7), Strep. agalactiae (15)*
16	557	11	0.1	16	—	16	16	—

Management and Environmental Factors

Current study revealed sand based stall systems along with sand or sawdust bedding as farm management (Supplementary Table 1). While the farm hygiene was evaluated on scale of 1 to 5 and ranged from 2-5, demonstrating variation in whole farm hygiene. The herds in all farms comprised of mainly Holstein-Friesian (HF) cows from different origins.

Supplementary Table 1. Management and Environmental Factors Associated with Mastitis Prevalence

Farm	Hygiene Score (1–5)	Stall Type	Bedding Type	Breed	Location
1	4	Sand	Sawdust	HF (US)	Gujranwala
2	3	Sand	Sand	HF (AUS)	Gujranwala
3	5	Sand	Sawdust	Cross-bred HF (AUS)	Sheikhupura
4	3	Sawdust	Sand	HF (AUS)	Sheikhupura
5	4	Sand	Sand	HF (AUS)	Narowal
6	2	Sand	Sand	HF (AUS)	Sahiwal
7	4	Sand	Sawdust	HF (AUS)	Lahore
8	4	Sand	Sand	HF (US)	Nankana Sahib
9	3	Sand	Sand	HF (Dutch)	Lahore
10	3	Sawdust	Sawdust	HF (AUS)	Kasur
11	4	Sand	Sand	HF (US)	Kasur
12	4	Sand	Sand	HF (AUS)	Sahiwal

13	4	Sawdust	Sawdust	HF (AUS)	Kasur
14	4	Sand	Sand	HF (AUS)	Faisalabad
15	3	Sand	Sand	HF (US)	Sahiwal
16	3	Sand	Sand	HF (US, AUS)	Kasur

Discussion

The empirical use of antibiotics in livestock is a recognized contributor to the global challenge of AMR. In dairy farming, clinical mastitis represents a primary reason for antimicrobial administration [21, 22]. Our randomized controlled trial demonstrates that a culture-based treatment strategy for CM, guided by a rapid on-farm tri-plate system, significantly reduces antibiotic use while maintaining clinical cure rates. This finding provides robust, practical evidence for implementing targeted antimicrobial stewardship (AMS) in commercial dairy operations.

The most direct impact of the CBG protocol was the substantial reduction in antibiotic use. By correctly identifying the 39.1% of cases with no bacterial growth and the 6.5% with Gram-negative growth, the protocol directed nearly half (45.6%) of all CBG cases to supportive care without antibiotics. This strategic withholding is a cornerstone of effective AMS. For Gram-negative pathogens like *Escherichia coli*, mastitis infections are often self-limiting, and the host's innate immune response is frequently effective at bacterial clearance [23]. Administering antibiotics in such cases offers limited clinical benefit while exerting unnecessary selection pressure for resistance. Our results provide field-based validation that this principle, previously demonstrated in controlled studies [17, 24, 25] can be operationalized effectively on farms to align with international AMS guidelines [26].

A critical finding was that this reduction in antibiotic use was achieved without compromising treatment efficacy. We found no significant difference between the CBG and PCT groups in the primary outcomes of clinical cure (72.8% vs. 75.4%, p=0.329) and bacteriological cure (71.8% vs. 71.9%, p=0.577). This non-inferiority is consistent with previous research reporting

maintained clinical outcomes while reducing antibiotic use by 40-50% through culture-guided decisions [17, 27, 28]. It robustly confirms that blanket therapy for all CM cases is an outdated and unnecessary practice.

For instance, CBG protocol demonstrated significant clinical advantages. The CBG approach resulted in a markedly lower treatment failure rate (19.35% vs. 80.65%, $p<0.001$) and a faster median time to clinical cure (3 days vs. 7 days). This suggests that targeted therapy, informed by a rapid 24-hour diagnosis, facilitates a more rapid and definitive resolution of infection. The faster recovery likely stems from ensuring the appropriate therapy is applied against the causative pathogen without delay, rather than relying on a broad-spectrum empirical approach that may be ineffective against the specific causative agent. In this regard our findings are in agreement with previous studies [27, 29].

Moreover, CM etiology confirmed from OFC system different pathogenic bacterial including highest incidence of gram-positive (45.7%, *Staphylococcus aureus*, *Streptococcus* spp., etc), followed by mixed infection (8.7%), and gram negative (6.5%) in CBG, our findings are highly consistent with previous studies [30, 31]. Nevertheless, the inconsistency in prevalence and pathogen profile across all the farms underscores the imperative need to have knowledge specific to the farm. The management data that includes the introduction of sand-based stalls and the variations in the hygienic scores reveal that the environmental factors were likely factors contributing to the risk of mastitis.

In the herd level, significant improvement was noticed in udder health after treatment. A significant reduction was noticed in the bulk tank SCC 159,859.2 ±105,334.1 post-treatment is crucial findings of our study. These values reduced significantly from a common industrial threshold of 200,000 cells/mL, which indicate good udder health. Consequently, this alleviation reduced the level of intra-mammary inflammation and mitigated bacterial load within the herd. Parallel to udder health, our study revealed significant increase in milk yield (32.56± 5.18, +6.94 L/day). As the inflammation reduces (reflect SCC reduction), secretary and production capability in the mammary gland restored, followed by milk synthesis and good quality. The findings of our study strongly consistent with previous studies [32, 33]. Several studies argued the inverse relationship between milk yield and SCC severe CM, where higher SCC level constantly reduced milk production [17, 34, 35].

The successful implementation of the tri-plate on-farm culture system was fundamental to the outcomes of this study. This system provided a pragmatic and cost-effective diagnostic solution, enabling pathogen-based therapeutic decisions within a clinically relevant timeframe. The design, incorporating Mannitol Salt Agar, Blood Agar, and MacConkey Agar, allowed for effective differentiation between major pathogen categories directly on the farm. Moreover, accomplishment of the CBG method in attaining these findings with fast clinical cure and rational antibiotic use directly correlates with principles of antimicrobial stewardship and targeted therapy. Our findings in this context supports previous authors [36], who established that using OFC guidelines to selective treatment for CM sustains equivalent clinical cure rates but also low decreases antibiotics usage to considerable amount (40-50%), irrespective negative impacts on cow health and milk yield. These findings robustly support the principle that pathogen-based decision-making is a viable and responsible practice. However, the field of on-farm diagnostics is not a single diagnostic standard; the performance, practicality, and specific clinical outcomes can vary considerably depending on the design of the system itself. Each unique platform, with its specific combination of media, incubation requirements, and interpretation criteria, requires independent and rigorous evaluation under field conditions to confirm its efficacy and practicality for farmers and veterinarians.

Future studies with larger, multi-regional cohorts would strengthen these findings. Furthermore, the operational efficacy of the on-farm culture system depends on proper training in aseptic sampling and plate interpretation. Future research should also investigate the long-term economic impact of this approach, balancing the initial diagnostic costs against savings from reduced antibiotic use, lower milk discard, and improved herd productivity.

Conclusions

This research provides compelling evidence that a rapid, on-farm tri-plate culture system can successfully guide a selective treatment protocol for clinical mastitis. This approach is a viable and superior alternative to immediate blanket antibiotic therapy, delivering equivalent primary cure rates while significantly accelerating recovery, reducing treatment failures, and advancing the critical goal of responsible antimicrobial use in dairy production.

Acknowledgments

This research was supported by the Punjab Agricultural Research Board (PARB) under the Competitive Grants System (Project ID No. 21-39).

References

1. Pakistan, G.o., *Pakistan Economic Survey 2024-25* 2025.
2. Mathew, M., et al., *Development of Zinc Oxide Nanoparticle and Lemongrass Essential Oil Incorporated Nanoformulation-Based External Teat Sealant to Prevent Mastitis in Dairy Cows.* 2025. **15**(1): p. 204.
3. Cheng, W.N. and S.G. Han, *Bovine mastitis: risk factors, therapeutic strategies, and alternative treatments - A review.* Asian-Australas J Anim Sci, 2020. **33**(11): p. 1699-1713.
4. Tong, X., et al., *Virulence of Bacteria Causing Mastitis in Dairy Cows: A Literature Review.* 2025. **13**(1): p. 167.
5. Ajose, D.J., et al., *Combating bovine mastitis in the dairy sector in an era of antimicrobial resistance: ethno-veterinary medicinal option as a viable alternative approach.* 2022. **9**: p. 800322.
6. Sharun, K., et al., *Advances in therapeutic and managemental approaches of bovine mastitis: a comprehensive review.* 2021. **41**(1): p. 107-136.
7. Ghumman, N.Z., et al., *Bovine mastitis and antimicrobial resistance in Pakistan's dairy sector: current status and future prospects.* Veterinary research communications, 2026. **50**(1): p. 1-23.
8. Roberts, J.J.L., *Selective treatment of clinical mastitis in dairy cows.* Livestock, 2025. **30**(Sup2): p. S12-S20.
9. Cha, E., et al., *The value of pathogen information in treating clinical mastitis.* Journal of Dairy Research, 2016. **83**(4): p. 456-463.
10. Sharun, K., et al., *Advances in therapeutic and managemental approaches of bovine mastitis: a comprehensive review.* Veterinary Quarterly, 2021. **41**(1): p. 107-136.
11. Zadoks, R., et al., *A framework for evaluation of on-farm mastitis diagnostics in Australia.* Australian Veterinary Journal, 2023. **101**(4): p. 142-152.
12. Neculai-Valeanu, A.-S. and A.-M.J.B. Ariton, *Udder health monitoring for prevention of bovine mastitis and improvement of milk quality.* Bioengineering, 2022. **9**(11): p. 608.
13. Saila, S., et al., *Evaluation of an on-farm culture system for the detection of subclinical mastitis pathogens in dairy cattle.* 2023. **4**(4): p. 298-302.
14. Tommasoni, C., et al., *Mastitis in dairy cattle: on-farm diagnostics and future perspectives.* Animals, 2023. **13**(15): p. 2538.
15. Ferreira, J.C., et al., *Comparative analysis of four commercial on-farm culture methods to identify bacteria associated with clinical mastitis in dairy cattle.* PLoS One, 2018. **13**(3): p. e0194211.
16. Ferreira, J.C., et al., *Comparative analysis of four commercial on-farm culture methods to identify bacteria associated with clinical mastitis in dairy cattle.* 2018. **13**(3): p. e0194211.
17. Lago, A., et al., *The selective treatment of clinical mastitis based on on-farm culture results: I. Effects on antibiotic use, milk withholding time, and short-term clinical and bacteriological outcomes.* Journal of dairy science, 2011. **94**(9): p. 4441-4456.

18. Royster, E., et al., *Evaluation of the Minnesota Easy Culture System II Bi-Plate and Tri-Plate for identification of common mastitis pathogens in milk.* Journal of dairy science, 2014. **97**(6): p. 3648-3659.
19. Ganda, E.K., et al., *Evaluation of an on-farm culture system (Accumast) for fast identification of milk pathogens associated with clinical mastitis in dairy cows.* PloS one, 2016. **11**(5): p. e0155314.
20. Council, N.M., *Laboratory Handbook on bovine mastitis*. 2005: NMC.
21. Shoaib, M., et al., *Emerging threat of antimicrobial resistance determinants and plasmid replicon types acquisition by Escherichia coli of poultry and other food-producing animal origin in China: local findings with global implications.* Poultry Science, 2025: p. 106055.
22. Li, X., et al., *Alternatives to antibiotics for treatment of mastitis in dairy cows.* Frontiers in veterinary science, 2023. **10**: p. 1160350.
23. Alhassan, M.Y., et al., *Revisiting antibiotic stewardship: veterinary contributions to combating antimicrobial resistance globally.* Bulletin of the National Research Centre, 2025. **49**(1): p. 25.
24. Sipka, A., et al., *Short communication: Comparative performance of 3 on-farm culture systems for detection of mastitis pathogens interpreted by trained and untrained observers.* Journal of Dairy Science, 2021. **104**(4): p. 4936-4941.
25. Auer, C., et al., *Training, development, and evaluation of on-farm culturing as a technical resource to reduce the use of antimicrobials in dairy herds in the Midwest Region of Santa Catarina State, Brazil.* Ciência Rural, 2021. **52**: p. e20200893.
26. Raka, L. and A. Kurti, *Addressing barriers in antimicrobial stewardship: a guide from research to implementation.* Journal of Global Antimicrobial Resistance, 2024. **39**: p. 26.
27. Borchardt, S. and W. Heuwieser, *Comparison of immediate blanket treatment versus a delayed pathogen-based treatment protocol for clinical mastitis using an on-farm culture test at a commercial German dairy farm.* Antibiotics, 2022. **11**(3): p. 368.
28. Breen, J., *Treating clinical mastitis in dairy herds: a role for on farm culture?* Livestock, 2021. **26**(5): p. 233-238.
29. Griffioen, K., et al., *Effects of a mastitis treatment strategy with or without on-farm testing.* Journal of Dairy Science, 2021. **104**(4): p. 4665-4681.
30. De Jong, E., *Enhancing Antimicrobial Stewardship On Dairy Farms With A Focus On Selective Treatment Of Clinical Mastitis.* 2024.
31. Sipka, A., et al., *Comparative performance of 3 on-farm culture systems for detection of mastitis pathogens interpreted by trained and untrained observers.* Journal of Dairy Science, 2021. **104**(4): p. 4936-4941.
32. Rowe, S., et al., *Randomized controlled trial investigating the effect of 2 selective dry-cow therapy protocols on udder health and performance in the subsequent lactation.* Journal of dairy science, 2020. **103**(7): p. 6493-6503.
33. Cortinhas, C.S., et al., *The association between socioeconomic profiles, attitudes, and knowledge of dairy farmers regarding somatic cell count and milk quality.* Animals, 2023. **13**(17): p. 2787.
34. Hadrich, J., et al., *Estimating milk yield and value losses from increased somatic cell count on US dairy farms.* Journal of dairy science, 2018. **101**(4): p. 3588-3596.
35. Danieli, B., et al., *Cows with High SCC Exhibit Poorer Performance and Milk Quality, Regardless of the Season.* Dairy, 2025. **6**(4): p. 46.
36. Lago, A. and S.M. Godden, *Use of rapid culture systems to guide clinical mastitis treatment decisions.* Veterinary Clinics: Food Animal Practice, 2018. **34**(3): p. 389-412.
