# Supplementary material for: Evaluation of tri-plate rapid on-farm culture system to make therapeutic decisions for mastitis cases in dairy cattle
Source: PLoS One. 2026 Jul 9;21(7):e0353527. doi: 10.1371/journal.pone.0353527 (PMC13349151; doi:10.1371/journal.pone.0353527)
Supplement: Attachment — Submitted filename: Recommendations.pdf [file pone.0353527.s005.pdf]

Recommendation

This study evaluates the efficacy of a Tri-plate on-farm culture (OFC) system in guiding therapeutic decisions for clinical mastitis in dairy cattle in Pakistan. The manuscript concludes that the CBG approach significantly reduced antibiotic use without compromising cure rates and improved herd-level metrics like milk yield and somatic cell count (SCC). While the study addresses a critical issue, antimicrobial stewardship in low-resource dairy settings and presents a valuable dataset, there are fundamental flaws in the statistical analysis and data presentation that must be resolved before publication. Specifically, the calculation of percentages in the outcome tables appears to represent the distribution of the total pool rather than the incidence rates within groups, leading to misleading conclusions in the abstract and results.

1. Critical Error in Statistical Reporting (Table 3)

The reporting of percentages in Table 3 and the abstract is mathematically incorrect and misleading. In Table 3, the authors report Treatment Failure as 80.65% for PCT and 19.35% for CBG. These two numbers sum to 100%. This indicates the authors calculated the *column percentage* (what portion of total failures belonged to each group) rather than the *row percentage* (what portion of the specific group failed). The abstract claims a significantly lower treatment failure rate (19.35% vs. 80.65%. This implies that 80% of PCT cows failed treatment, which contradicts the claim in the same sentence that cure rates were non-inferior. The authors must recalculate *all* percentages in Table 3 (Clinical Cure, Bacteriological Cure, etc.) to reflect the incidence rate *within the treatment arm*, not the share of the total study population. The statistical tests (Chi-square) must be re-run on the raw counts to ensure the p-values remain valid.

2. The study enrolled 103 cows but discusses 151 quarters. It is unclear if the randomization and treatment were applied at the *cow level* or the *quarter level*. If a cow had two infected quarters, was she treated as one unit, or were quarters treated differently?

If multiple quarters within the same cow were included, the observations are not independent. Standard Chi-square and t-tests are inappropriate for clustered data. The authors should use mixed-effects models (accounting for clustering of quarters within cows, and cows within herds) or strictly limit the analysis to one quarter per cow. The Demographic Characteristics table lists Number of Infected Quarters per cow, suggesting clustering is present.

3. Clarification of Clinical Cure vs. Treatment Failure

In Table 3, Clinical Cure and Treatment Failure do not seem to align with the raw N values.

PCT Clinical Cure: 43 cows.

PCT Treatment Failure: 25 cows.

Total PCT: 43 + 25 = 68. However, the total PCT sample size is listed as n=57.

The numbers do not sum correctly to the sample size. The authors must audit their data. Is there an overlap between categories? Were cows lost to follow-up? The definitions of Cure and Failure must be mutually exclusive or clearly defined if they overlap.

4. Kaplan-Meier Analysis (Figure 1)

The survival curves in Figure 1 show precipitous drops at exactly Day 3 and Day 7. This suggests the Time to Cure was not measured continuously but rather assessed at specific protocol intervals (e.g., end of the 3-day treatment). The authors should clarify the observation frequency in the methods. If cows were only checked on Day 3 and Day 7, this should be stated as interval-censored data, or the limitations of the precision of days to cure should be discussed.

5. Sample Size and Randomization

The text states, formal sample size calculation was not conducted and quarters were randomly allocated.

While *PLOS ONE* accepts technically sound negative or pilot results, the lack of power calculation limits the ability to claim non-inferiority definitively. More importantly, the authors must describe the *method* of randomization (e.g., random number generator, sealed envelopes). Randomly allocated is insufficient detail for an RCT.

Minor Revisions

Typographical and Grammatical Errors: The manuscript requires thorough copy-editing.

Dairy Cattles should be Dairy Cattle.

Tom accomplish this should be To accomplish this.

fro clinically effected should be from clinically affected.

Author names and affiliations need consistent formatting.

Reference Formatting: The bibliography is inconsistent. Some references have full journal titles, others abbreviated; capitalization varies.

Inconsistent N values: The abstract mentions PCT n=57 and CBG n=46. However, the results text mentions 57 cows with 83 effected quarters and 46 cows with 68 effected. Please ensure n-values are consistent throughout the text and tables.

Funding Statement: The cover letter and manuscript body acknowledge funding from PARB, but the authors should ensure this is consistent with the metadata entered in the submission system.

Recommendation regarding References: To strengthen the context regarding antimicrobial resistance (AMR) and environmental pathogens, the authors might consider and cite the following recent studies:

1. Zhou, L., Ou, S., Liang, T., Li, M., Xiao, P., Cheng, J., Zhou, J., & Yuan, L. (2023). MAEL facilitates metabolic reprogramming and breast cancer progression by promoting the degradation of citrate synthase and fumarate hydratase via chaperone-mediated autophagy. The FEBS journal, 290(14), 3614–3628. https://doi.org/10.1111/febs.16768
2. Hu, Y. L., Zhang, Y. J., Lv, X. Y., Liu, R. L., Zhong, Z. H., Fu, L. J., Bao, M. H., Geng, L. H., Xu, H. J., Yu, S. M., & Ding, Y. B. (2024). Impact of Omicron Variant Infection on Female Fertility and Laboratory Outcomes: A Self-Controlled Study. American journal of reproductive immunology (New York, N.Y. : 1989), 92(5), e70012. https://doi.org/10.1111/aji.70012
3. Kang, S., Jin, S., Mao, X., He, B., & Wu, C. (2024). CD4+T and CD8+T Cells in Uterus Exhibit Both Selective Dysfunction and Residency Signatures. Journal of Immunology Research, 2024(1), 5582151. doi: https://doi.org/10.1155/2024/5582151
4. Yao, Y. L., Xu, Z., Xiao, R., Li, E. H., Zhang, Y. J., Zhou, L. Y., Zhong, Z. H., Fu, L. J., Qi, H. B., Wu, X. B., & Ding, Y. B. (2025). Elevated Neutrophil, Lymphocyte, and Platelet Counts as Early Biomarkers of Preeclampsia Risk: A Retrospective Cohort Study. American journal of reproductive immunology (New York, N.Y. : 1989), 94(2), e70137. https://doi.org/10.1111/aji.70137
5. Gohar, S., Abbas, G., & Sanaullah, S. (2017). Prevalence and antimicrobial resistance of Listeria monocytogenes isolated from raw milk and dairy products. Zibeline International Publishing, 1(1), 10-14.

6. Nayab, S., ur Rahman, S., Sajid, S., & Abbas, M. I. (2022). Efficacy of Allicin against multi-drug resistant Escherichia coli recovered from potable water. Pure and Applied Biology, 11(2), 505-513.
