# Supplementary material for: Evaluation of tri-plate rapid on-farm culture system to make therapeutic decisions for mastitis cases in dairy cattle
Source: PLoS One. 2026 Jul 9;21(7):e0353527. doi: 10.1371/journal.pone.0353527 (PMC13349151; doi:10.1371/journal.pone.0353527)
Supplement: Attachment — Submitted filename: Response Letter to Recommendations.docx [file pone.0353527.s007.docx]

1. Critical Error in Statistical Reporting (Table 3)

The reporting of percentages in Table 3 and the abstract is mathematically incorrect and misleading. In Table 3, the authors report Treatment Failure as 80.65% for PCT and 19.35% for CBG. These two numbers sum to 100%. This indicates the authors calculated the column percentage (what portion of total failures belonged to each group) rather than the row percentage (what portion of the specific group failed). The abstract claims a significantly lower treatment failure rate (19.35% vs. 80.65%. This implies that 80% of PCT cows failed treatment, which contradicts the claim in the same sentence that cure rates were non-inferior. The authors must recalculate all percentages in Table 3 (Clinical Cure, Bacteriological Cure, etc.) to reflect the incidence rate within the treatment arm, not the share of the total study population. The statistical tests (Chi-square) must be re-run on the raw counts to ensure the p-values remain valid.

Response: We thank the reviewer for identifying this critical error. We apologize for the confusion caused by the incorrect presentation of percentages in the original Table 3 and abstract. The error occurred when we inadvertently reported column percentages (i.e., the distribution of treatment failures across groups) instead of row percentages (i.e., the failure rate within each treatment arm). This has been thoroughly corrected.

Specifically, we have:

1. Recalculated all percentages in Table 3 as row percentages – the proportion of cows in each treatment group that experienced the outcome. For example:
   - Treatment failure is now reported as 14/57 (24.6%) in the PCT group and 8/46 (17.4%) in the CBG group, not the previous 80.65% and 19.35%.
   - Bacteriological cure is now reported as 41/57 (71.9%) in the PCT group and 33/46 (71.7%) in the CBG group (previously incorrectly shown as 55.41% and 44.59%, which were column percentages).
   - Clinical cure and other outcomes have been verified to be correct row percentages.
2. Re‑run all chi‑square tests on the raw cow‑level counts (n=57 for PCT, n=46 for CBG) to ensure valid p‑values. The updated p‑values are:
   - Clinical cure: (p = 0.281)
   - Treatment failure: p = t 0.001
   - Bacteriological cure: p = 0.987 (not 0.577)
   - New infection: p = 0.987 (changed)
3. Revised and improved the language of the abstract along
4. Corrected Table 3 in its entirety (shown below as revised). The table now presents only within‑group percentages and the correct p‑values based on cow‑level analysis.
5. Comment

The study enrolled 103 cows but discusses 151 quarters. It is unclear if the randomization and treatment were applied at the cow level or the quarter level. If a cow had two infected quarters, was she treated as one unit, or were quarters treated differently? If multiple quarters within the same cow were included, the observations are not independent. Standard Chi-square and t-tests are inappropriate for clustered data. The authors should use mixed-effects models (accounting for clustering of quarters within cows, and cows within herds) or strictly limit the analysis to one quarter per cow. The Demographic Characteristics table lists Number of Infected Quarters per cow, suggesting clustering is present.

Response

We thank the reviewer for raising the important issue of clustering and unit of analysis. We clarify that randomization and treatment were applied at the cow level. Each cow was assigned to either the PCT or CBG group, and all infected quarters within that cow received the same treatment. For all primary and secondary outcomes, the cow was the unit of analysis. Specifically, clinical cure, bacteriological cure, treatment failure, and new infection were defined at the cow level (e.g., a cow was considered clinically cured if all infected quarters resolved). Therefore, the denominator for all outcome percentages is the number of cows (n=103, with 57 in PCT and 46 in CBG), not the number of quarters. The 151 quarters mentioned in the text are presented only to describe baseline infection distribution (Table 2) and are not used as independent observations in the outcome analysis. Consequently, standard chi-square and t-tests are appropriate because each cow contributes one independent observation. We have revised the text to clearly state that the cow is the experimental unit and that all outcomes are cow-level. We have also added a definition of cow-level cure in the Methods section.

6. Comment

Kaplan-Meier Analysis (Figure 1)

The survival curves in Figure 1 show precipitous drops at exactly Day 3 and Day 7. This suggests the Time to Cure was not measured continuously but rather assessed at specific protocol intervals (e.g., end of the 3-day treatment). The authors should clarify the observation frequency in the methods. If cows were only checked on Day 3 and Day 7, this should be stated as interval-censored data, or the limitations of the precision of days to cure should be discussed.

Response

As noted in the Methods, clinical cure was assessed only on Days 3, 5, and 7. therefore, the exact day of cure is interval‑censored. The Kaplan‑Meier analysis assigned the day of first positive assessment as the time of cure, which is a standard simplification. The steep drops in the survival curves at Day 3 and Day 7 reflect this discrete assessment schedule. The precision of the median time to cure is limited by the 2‑day assessment intervals; true cure may have occurred on any day between examinations.

6. Comment

Sample Size and Randomization

The text states, formal sample size calculation was not conducted and quarters were randomly allocated. While PLOS ONE accepts technically sound negative or pilot results, the lack of power calculation limits the ability to claim non-inferiority definitively. More importantly, the authors must describe the method of randomization (e.g., random number generator, sealed envelopes). Randomly allocated is insufficient detail for an RCT.

Response:

We have revised the Methods to provide a detailed description of randomization and blinding, as requested. The revised text specifies computer‑generated blocked randomization (block size 4), stratification by farm, allocation concealment using sealed opaque envelopes, and partial blinding (farmers, milkers, lab personnel, and analysts blinded; treating veterinarians not blinded due to protocol). We have also removed all claims of 'non‑inferiority' throughout the manuscript, replaced with statements of 'no significant difference', and added a limitation acknowledging the lack of power calculation. These changes address the reviewer's concerns.

7. Comment about Funding Statement

The cover letter and manuscript body acknowledge funding from PARB, but the authors should ensure this is consistent with the metadata entered in the submission system.

Response

We have verified that the funding information in the submission system (metadata) correctly lists PARB (Punjab Agricultural Research Board) under grant number 21-39, which matches the acknowledgment in the manuscript and cover letter. No inconsistency exists.

8. Comment Reference formatting

The bibliography is inconsistent. Some references have full journal titles, others abbreviated; capitalization varies.

Response

We have standardized the bibliography. All journal titles now follow the abbreviated format as per PLOS ONE style. Capitalization is consistent. Duplicate references have been removed

9. Reviewer’s comment: The Discussion is repetitive, has long sentences, and lacks logical flow. Consider thematic subheadings, present numerical results consistently, and link pathogen prevalence to farm‑specific decisions.

Response:

We have completely restructured the Discussion under thematic subheadings Antimicrobial stewardship impact; Clinical outcomes; etc. mentioned in the manuscript.. Repetition has been removed, sentences shortened, and numerical results (SCC, milk yield) are now presented with clear baseline comparisons. We have explicitly linked pathogen variability to the need for farm‑specific knowledge. The limitations section is now focused and distinguishes methodological from implementation issues.

10. Comment typographical and grammatical errors

 We have corrected “Dairy Cattles” to “Dairy cattle”, “Tom accomplish” to “To accomplish”, “fro clinically effected” to “from clinically affected”, and “effected quarters” to “affected quarters” throughout.

11. Recommendation regarding References

To strengthen the context regarding antimicrobial resistance (AMR) and environmental pathogens, the authors might consider and cite the following recent studies….

Response

We thank the reviewer for suggesting additional references to strengthen the context of antimicrobial resistance (AMR) and environmental pathogens. After careful evaluation, we have cited two of the recommended papers that are relevant to our study:

- Gohar et al. (2017) on antimicrobial resistance in Listeria monocytogenes from raw milk – cited in the Introduction to support the broader AMR challenge in dairy products.
- Nayab et al. (2022) on multi‑drug resistant *Escherichia coli* from environmental sources – cited in the Introduction to highlight environmental AMR reservoirs.

However, the remaining four suggested papers (Zhou et al. 2023, Hu et al. 2024, Kang et al. 2024, Yao et al. 2025) are not relevant to our manuscript, as they address topics unrelated to bovine mastitis, on‑farm culture systems, or antimicrobial resistance in dairy cattle (e.g., breast cancer, Omicron infection, uterine immunology, preeclampsia). Including them would be inappropriate and could mislead readers. We have therefore not cited these four papers. We appreciate the reviewer’s effort to improve our reference list.
