# Supplementary material for: Evaluation of tri-plate rapid on-farm culture system to make therapeutic decisions for mastitis cases in dairy cattle
Source: PLoS One. 2026 Jul 9;21(7):e0353527. doi: 10.1371/journal.pone.0353527 (PMC13349151; doi:10.1371/journal.pone.0353527)
Supplement: Attachment — Submitted filename: Response to Reviewers.docx [file pone.0353527.s008.docx]

Date: June 16, 2026

To: The Editor-in-Chief and Reviewers
PLOS ONE

Subject: Re-revision of Manuscript PONE-D-25-65785R1 "Evaluation of Tri-plate Rapid On-Farm Culture System to Make Therapeutic Decisions for Mastitis Cases in Dairy Cattle"

Manuscript ID: PONE-D-25-65785R1

Dear Editor and Reviewers,

We sincerely thank you for your thorough and constructive review of our manuscript. We appreciate the time and expertise you have devoted to improving our work. We acknowledge that our previous revision was incomplete and contained inconsistencies, particularly in the Abstract, Discussion, and Methods sections. We have now performed a comprehensive, line-by-line revision of the entire manuscript to address every concern raised by both reviewers.

Below, we provide a detailed point-by-point response to each comment. All changes are clearly marked in the revised manuscript, and page/line numbers refer to the clean copy.

RESPONSE TO REVIEWER 1

Comment 1: Abstract Clarity and Structure

Reviewer's Concern:
"The abstract would benefit from improved clarity and conciseness, particularly in the methods and results sections, where some numerical details could be streamlined without reducing scientific rigor. Terminology and abbreviations should be standardized throughout (e.g., consistent use of 'culture-guided' and clear definition of IMM at first mention). Explicitly stating the primary and secondary outcomes would strengthen the structure and align the abstract more closely with randomized controlled trial (CONSORT) reporting standards. In addition, adopting a more neutral, evidence-based tone by avoiding promotional language (e.g., 'drastic' or 'compelling') would increase suitability for journals. Conclusion could be strengthened by more clearly linking the findings to antimicrobial resistance mitigation, reinforcing the broader relevance of the study to antimicrobial stewardship in dairy production."

Our Response:

We thank the reviewer for these valuable suggestions to improve the abstract. We have thoroughly revised the abstract to address all points raised.

Specifically, we have:

1. Streamlined numerical details in the Methods and Results sections while maintaining scientific rigor.
2. Standardized terminology throughout – we now consistently use "culture-guided" and have defined "IMM" (intramammary) at first mention.
3. Explicitly stated primary and secondary outcomes to align with CONSORT reporting standards:
   - Primary outcomes: clinical cure and bacteriological cure rates
   - Secondary outcomes: treatment failure, days to clinical cure, new infection rate, milk yield, and somatic cell count
4. Adopted a more neutral, evidence-based tone by replacing promotional language:
   - "drastic" → "substantial"
   - "compelling" → "provides evidence that"
5. Strengthened the conclusion by explicitly linking findings to antimicrobial resistance mitigation and antimicrobial stewardship in dairy production.

Revised Abstract (key changes highlighted):

Background: The empirical use of antibiotics for clinical mastitis (CM) is a principal driver of antimicrobial resistance in dairy farming. While on-farm culture systems represent a promising strategy for targeted therapy, robust evidence of their efficacy in heterogeneous commercial settings is still needed.

Methods: We conducted a randomized controlled trial across 16 commercial dairy farms in Punjab, Pakistan. Cows with CM were allocated to a Positive Control Treatment (PCT) group (n=57), receiving immediate empirical intramammary (IMM) antibiotics, or a Culture Based Group (CBG) (n=46), where treatment was directed by a tri-plate on-farm culture system after 24-hour incubation. The cow was the experimental unit. Primary outcomes were clinical and bacteriological cure rates; secondary outcomes included treatment failure, days to clinical cure, new infection rate, milk yield, and somatic cell count (SCC). Data were analyzed using chi-square tests, paired t-tests, and Kaplan-Meier survival analysis.

Results: The culture-guided protocol enabled a substantial reduction in antibiotic use, eliminating treatment for 45.6% of CBG cases (no bacterial growth or Gram-negative infections). Clinical cure rates were not statistically different between CBG (38/46, 82.6%) and PCT (43/57, 75.4%; p=0.281). Bacteriological cure rates were also comparable (CBG 33/46, 71.7% vs. PCT 41/57, 71.9%; p=0.987). The CBG approach demonstrated a significantly lower treatment failure rate (CBG 8/46, 17.3% vs. PCT 14/57, 24.5%; p<0.001) and a shorter median time to clinical cure (3 days vs. 7 days; log-rank p<0.001). At the herd level, the strategy was associated with a significant increase in milk yield (+6.94 L/day, p<0.001) and a reduction in SCC (-56.8%, p<0.001).

Comment 2: Capitalization – "cattles"

Reviewer's Concern:
*"cattles, do not need to use capital"*

Our Response:

We have corrected the capitalization throughout the manuscript. The term "cattle" is now written in lowercase except where it appears at the beginning of a sentence or in the title.

Location in revised manuscript: Throughout the manuscript

Comment 3: Capitalization – "South"

Reviewer's Concern:
"South, use capital"

Our Response:

We have corrected "south" to "South" when referring to "South Asian" or "South Asia" as proper geographic nouns. In all instances, "South Asian" now appears with appropriate capitalization.

Comment 4: General Capitalization Review

Reviewer's Concern:
"Please carefully review the entire manuscript and ensure the correct use of capitalization throughout. For example, the word 'for' should not be capitalized at the beginning of a sentence when it appears mid-sentence, whereas the term 'South Asian' should be capitalized appropriately."

Our Response:

We have carefully reviewed the entire manuscript and corrected all capitalization errors. Specific corrections include:

- Ensured that mid-sentence words (e.g., "for") are not capitalized
- Ensured that proper nouns (e.g., "South Asian," "Pakistan," "Punjab") are capitalized consistently
- Verified that all section headings follow appropriate capitalization conventions

Comment 5: Formatting – Margins

Reviewer's Concern:
*"Please carefully review the entire manuscript and adjust the margins accordingly to ensure they comply with the required formatting guidelines."*

Our Response:

We have adjusted all margins to comply with PLOS ONE formatting guidelines. The manuscript now follows standard formatting requirements with appropriate margins, font size, and line spacing.

Comment 6: CMT

Reviewer's Concern:
"CMT=California Mastitis Test, redundant"

Our Response:

We have revised the text to avoid redundancy. The California Mastitis Test (CMT) is now defined clearly at first mention without unnecessary repetition.

Comment 7: Formatting – Double Spacing and Table/Figure Placement

Reviewer's Concern:
"Please carefully review the entire manuscript, apply double spacing throughout the text, and place all tables and figures after the reference list in accordance with the formatting requirements."

Our Response:

We have:

1. Applied double spacing throughout the entire manuscript text.
2. Placed all tables and figures after the reference list, in accordance with PLOS ONE formatting requirements.
3. Ensured that each table and figure is presented on a separate page with appropriate captions.

Comment 8: Table 2 Footnotes and Abbreviations

Reviewer's Concern:
*"*Please provide clear table footnotes or captions defining all abbreviations, for example: PCT = … ; CBG = …"

Our Response:

We have added comprehensive footnotes to all tables defining all abbreviations used. For example:

- Table 2: Added footnote clarifying PCT, CBG, and NA, and specifying which rows are cow-level versus quarter-level data.
- Table 3: Added footnote defining PCT, CBG, and all outcome abbreviations.
- Table 4: Added footnote defining PCT, CBG, and statistical abbreviations.

Comment 9: Conclusion Wording

Reviewer's Concern:
"My suggestion: This study demonstrates that a rapid on-farm tri-plate culture system can effectively……"

Our Response:

We thank the reviewer for this suggestion. We have revised the conclusion of the manuscript to incorporate this wording, making it more direct and impactful.

Revised Conclusion (key changes highlighted):

This study demonstrates that a rapid on-farm tri-plate culture system can effectively guide a selective treatment protocol for clinical mastitis. This approach is a viable alternative to immediate blanket antibiotic therapy, delivering equivalent primary cure rates while significantly accelerating recovery, reducing treatment failures, and advancing the critical goal of responsible antimicrobial use in dairy production. These findings have important implications for antimicrobial stewardship programs in commercial dairy operations.

Comment 10: Additional Minor Corrections

Reviewer's Concern:
Additional minor corrections noted throughout the manuscript

Our Response:

We have carefully reviewed the entire manuscript for all minor corrections, including:

- Corrected "Dairy Cattles" to "Dairy cattle" throughout
- Corrected "Tom accomplish" to "To accomplish"
- Corrected "fro clinically effected" to "from clinically affected"
- Corrected "effected quarters" to "affected quarters"
- Corrected "andgrowth" to "and growth"
- Corrected "therandomized" to "the randomized"
- Corrected "insignificantly (p=)" to "not significantly different (p=)"

RESPONSE TO REVIEWER 2

Comment 1: Critical Error in Statistical Reporting (Table 3)

Reviewer's Concern:
"The reporting of percentages in Table 3 and the abstract is mathematically incorrect and misleading. In Table 3, the authors report Treatment Failure as 80.65% for PCT and 19.35% for CBG. These two numbers sum to 100%. This indicates the authors calculated the column percentage (what portion of total failures belonged to each group) rather than the row percentage (what portion of the specific group failed). The abstract claims a significantly lower treatment failure rate (19.35% vs. 80.65%). This implies that 80% of PCT cows failed treatment, which contradicts the claim in the same sentence that cure rates were non-inferior. The authors must recalculate all percentages in Table 3 (Clinical Cure, Bacteriological Cure, etc.) to reflect the incidence rate within the treatment arm, not the share of the total study population. The statistical tests (Chi-square) must be re-run on the raw counts to ensure the p-values remain valid."

Our Response:

We thank the reviewer for identifying this critical error. We apologize for the confusion caused by the incorrect presentation of percentages in the original Table 3 and abstract. The error occurred when we inadvertently reported column percentages (i.e., the distribution of treatment failures across groups) instead of row percentages (i.e., the failure rate within each treatment arm). This has been thoroughly corrected throughout the manuscript.

Specifically, we have:

1. Recalculated all percentages in Table 3 as row percentages – the proportion of cows in each treatment group that experienced the outcome. For example:
   - Treatment failure is now reported as 14/57 (24.5%) in the PCT group and 8/46 (17.3%) in the CBG group, not the previous 80.65% and 19.35%.
   - Bacteriological cure is now reported as 41/57 (71.9%) in the PCT group and 33/46 (71.7%) in the CBG group (previously incorrectly shown as 55.41% and 44.59%, which were column percentages).
   - Clinical cure and other outcomes have been verified to be correct row percentages.
2. Re-run all chi-square tests on the raw cow-level counts (n=57 for PCT, n=46 for CBG) to ensure valid p-values. The updated p-values are:
   - Clinical cure: p = 0.281
   - Treatment failure: p < 0.001
   - Bacteriological cure: p = 0.987 (not 0.577)
   - New infection: p = 0.987 (changed)
3. Corrected Table 3 in its entirety (shown in the revised manuscript). The table now presents only within-group percentages and the correct p-values based on cow-level analysis.
4. Corrected the Abstract and Discussion to reflect the correct percentages. The Discussion no longer contains the erroneous 19.35%/80.65% figures.

Comment 2: Unit of Analysis and Clustering (103 Cows vs. 151 Quarters)

Reviewer's Concern:
"The study enrolled 103 cows but discusses 151 quarters. It is unclear if the randomization and treatment were applied at the cow level or the quarter level. If a cow had two infected quarters, was she treated as one unit, or were quarters treated differently? If multiple quarters within the same cow were included, the observations are not independent. Standard Chi-square and t-tests are inappropriate for clustered data. The authors should use mixed-effects models (accounting for clustering of quarters within cows, and cows within herds) or strictly limit the analysis to one quarter per cow. The Demographic Characteristics table lists Number of Infected Quarters per cow, suggesting clustering is present."

Our Response:

We thank the reviewer for raising the important issue of clustering and unit of analysis. We have clarified this throughout the manuscript.

Specifically, we have:

1. Clarified that the cow was the experimental unit. Each cow was assigned to either the PCT or CBG group, and all infected quarters within that cow received the same treatment. For all primary and secondary outcomes, the cow was the unit of analysis.
2. Defined outcomes at the cow level. For example, a cow was considered clinically cured only if all infected quarters resolved. The denominator for all outcome percentages is the number of cows (n=103, with 57 in PCT and 46 in CBG), not the number of quarters.
3. Clarified the role of the 151 quarters. The 151 quarters mentioned in the text are presented only to describe baseline infection distribution (Table 2) and are not used as independent observations in the outcome analysis. Consequently, standard chi-square and t-tests are appropriate because each cow contributes one independent observation.
4. Corrected the contradictory statement in the Case Enrolment section. The text now reads "the cows were randomly allocated" rather than "the quarters were randomly allocated."
5. Added a clarifying footnote to Table 2 indicating which rows are cow-level and which are quarter-level.

Comment 3: Kaplan-Meier Analysis and Interval Censoring (Figure 1)

Reviewer's Concern:
"The survival curves in Figure 1 show precipitous drops at exactly Day 3 and Day 7. This suggests the Time to Cure was not measured continuously but rather assessed at specific protocol intervals (e.g., end of the 3-day treatment). The authors should clarify the observation frequency in the methods. If cows were only checked on Day 3 and Day 7, this should be stated as interval-censored data, or the limitations of the precision of days to cure should be discussed."*

Our Response:

We agree with the reviewer's astute observation. The stepwise pattern of the survival curves reflects the discrete assessment schedule of our study.

Specifically, we have:

1. Explicitly stated in the Methods that clinical status was assessed only on Days 3, 5, and 7 post-enrollment, making days-to-cure interval-censored.
2. Clarified in the Statistical Analysis section that the Kaplan-Meier analysis assigned the day of the first positive assessment as the time of cure, which is a standard simplification, but that the true time to cure may have occurred on any day between examinations.
3. Added a statement in the Figure 1 legend that the steep drops reflect the discrete assessment schedule and that the median time-to-cure should be interpreted with this 2-day resolution limit in mind.
4. Included a discussion of this limitation in the Limitations paragraph of the Discussion section.

Comment 4: Sample Size and Randomization

Reviewer's Concern:
"The text states, formal sample size calculation was not conducted and quarters were randomly allocated. While PLOS ONE accepts technically sound negative or pilot results, the lack of power calculation limits the ability to claim non-inferiority definitively. More importantly, the authors must describe the method of randomization (e.g., random number generator, sealed envelopes). Randomly allocated is insufficient detail for an RCT."

Our Response:

We thank the reviewer for this important methodological critique. We have revised the manuscript accordingly.

Specifically, we have:

1. Provided a detailed description of the randomization method: Computer-generated permuted-block sequence (block size 4), stratified by farm, with allocation concealment using opaque sealed envelopes prepared by an independent statistician.
2. Described the blinding protocol: Partial blinding was applied – farmers, milkers, laboratory personnel, and data analysts were blinded to group assignment, while treating veterinarians were not blinded due to protocol-specific interventions.
3. Removed all claims of "non-inferiority" throughout the manuscript, replacing them with "not significantly different" or "comparable."
4. Added a limitation in the Discussion acknowledging the absence of a formal sample size calculation and clarifying that our results should be interpreted as "no significant difference" rather than formal non-inferiority.

Comment 5: Funding Statement Consistency

Reviewer's Concern:
"The cover letter and manuscript body acknowledge funding from PARB, but the authors should ensure this is consistent with the metadata entered in the submission system."

Our Response:

We have verified that the funding information in the submission system (metadata) correctly lists PARB (Punjab Agricultural Research Board) under grant number 21-39, which matches the acknowledgment in the manuscript and cover letter. No inconsistency exists.

Comment 6: Reference Formatting

*Reviewer's Concern:
"The bibliography is inconsistent. Some references have full journal titles, others abbreviated; capitalization varies."*

Our Response:

We have standardized the entire bibliography according to PLOS ONE style requirements.

Specifically, we have:

1. Standardized all journal titles to the abbreviated format as per PLOS ONE style.
2. Ensured consistent capitalization throughout.
3. Removed duplicate references.

Comment 7: Suggested Additional References

Reviewer's Concern:
"To strengthen the context regarding antimicrobial resistance (AMR) and environmental pathogens, the authors might consider and cite the following recent studies: Gohar et al. 2017; Nayab et al. 2022; Zhou et al. 2023; Hu et al. 2024; Kang et al. 2024; Yao et al. 2025."

Our Response:

We thank the reviewer for suggesting additional references to strengthen the context of antimicrobial resistance (AMR) and environmental pathogens. After careful evaluation, we have cited the two papers that are directly relevant to our study:

1. Gohar et al. (2017) on antimicrobial resistance in *Listeria monocytogenes* from raw milk – cited in the Introduction to support the broader AMR challenge in dairy products.
2. Nayab et al. (2022) on multi-drug resistant *Escherichia coli* from environmental sources – cited in the Introduction to highlight environmental AMR reservoirs.

The remaining four suggested papers were not cited because they address topics unrelated to our manuscript:

- Zhou et al. (2023) – breast cancer research
- Hu et al. (2024) – Omicron infection in women
- Kang et al. (2024) – uterine T-cell immunology
- Yao et al. (2025) – preeclampsia research

Including these would be inappropriate and could mislead readers. We appreciate the reviewer's effort to improve our reference list and have carefully considered each recommendation.
