# Supplementary material for: Evaluation of tri-plate rapid on-farm culture system to make therapeutic decisions for mastitis cases in dairy cattle
Source: PLoS One. 2026 Jul 9;21(7):e0353527. doi: 10.1371/journal.pone.0353527 (PMC13349151; doi:10.1371/journal.pone.0353527)
Supplement: Peer review history

# Peer review: Decision Letter 0

**DOI:** 10.1371/journal.pone.0353527.r001

**Authors:** Sanaullah Sajid

23 Jan 2026

PONE-D-25-65785

Evaluation of Tri-plate Rapid On-Farm Culture System to Make Therapeutic Decisions for Mastitis Cases in Dairy Cattle

PLOS One

Dear Dr. Sajjad Ahmad,

Thank you for submitting your manuscript to PLOS ONE. After careful consideration, we feel that it has merit but does not fully meet PLOS ONE’s publication criteria as it currently stands. Therefore, we invite you to submit a revised version of the manuscript that addresses the points raised during the review process.

Please submit your revised manuscript by Mar 09 2026 11:59PM. If you will need more time than this to complete your revisions, please reply to this message or contact the journal office at plosone@plos.org. When you're ready to submit your revision, log on to https://www.editorialmanager.com/pone/ and select the 'Submissions Needing Revision' folder to locate your manuscript file.

Please include the following items when submitting your revised manuscript:

- A letter that responds to each point raised by the academic editor and reviewer(s). You should upload this letter as a separate file labeled 'Response to Reviewers'.
- A marked-up copy of your manuscript that highlights changes made to the original version. You should upload this as a separate file labeled 'Revised Manuscript with Track Changes'.
- An unmarked version of your revised paper without tracked changes. You should upload this as a separate file labeled 'Manuscript'.

If you would like to make changes to your financial disclosure, please include your updated statement in your cover letter. Guidelines for resubmitting your figure files are available below the reviewer comments at the end of this letter.

If applicable, we recommend that you deposit your laboratory protocols in protocols.io to enhance the reproducibility of your results. Protocols.io assigns your protocol its own identifier (DOI) so that it can be cited independently in the future. For instructions see: https://journals.plos.org/plosone/s/submission-guidelines#loc-laboratory-protocols. Additionally, PLOS ONE offers an option for publishing peer-reviewed Lab Protocol articles, which describe protocols hosted on protocols.io. Read more information on sharing protocols at https://plos.org/protocols?utm_medium=editorial-email&utm_source=authorletters&utm_campaign=protocols.

We look forward to receiving your revised manuscript.

Kind regards,

Sanaullah Sajid, M.Phil/PhD

Academic Editor

PLOS One

Journal requirements:

When submitting your revision, we need you to address these additional requirements.

1. Please ensure that your manuscript meets PLOS ONE's style requirements, including those for file naming. The PLOS ONE style templates can be found at

https://journals.plos.org/plosone/s/file?id=wjVg/PLOSOne_formatting_sample_main_body.pdf and

https://journals.plos.org/plosone/s/file?id=ba62/PLOSOne_formatting_sample_title_authors_affiliations.pdf

2. Thank you for stating the following financial disclosure:

“This research was supported by the Punjab Agricultural Research Board (PARB) under the Competitive Grants System (Project ID No. 21-39).”

Please state what role the funders took in the study.  If the funders had no role, please state: "The funders had no role in study design, data collection and analysis, decision to publish, or preparation of the manuscript."

If this statement is not correct you must amend it as needed.

Please include this amended Role of Funder statement in your cover letter; we will change the online submission form on your behalf.

3. Thank you for stating the following in the Acknowledgments Section of your manuscript:

“This research was supported by the Punjab Agricultural Research Board (PARB) under the Competitive Grants System (Project ID No. 21-39).”

We note that you have provided funding information that is currently declared in your Funding Statement. However, funding information should not appear in the Acknowledgments section or other areas of your manuscript. We will only publish funding information present in the Funding Statement section of the online submission form.

Please remove any funding-related text from the manuscript and let us know how you would like to update your Funding Statement. Currently, your Funding Statement reads as follows:

“This research was supported by the Punjab Agricultural Research Board (PARB) under the Competitive Grants System (Project ID No. 21-39).”

Please include your amended statements within your cover letter; we will change the online submission form on your behalf.

4. PLOS requires an ORCID iD for the corresponding author in Editorial Manager on papers submitted after December 6th, 2016. Please ensure that you have an ORCID iD and that it is validated in Editorial Manager. To do this, go to ‘Update my Information’ (in the upper left-hand corner of the main menu), and click on the Fetch/Validate link next to the ORCID field. This will take you to the ORCID site and allow you to create a new iD or authenticate a pre-existing iD in Editorial Manager.

If the reviewer comments include a recommendation to cite specific previously published works, please review and evaluate these publications to determine whether they are relevant and should be cited. There is no requirement to cite these works unless the editor has indicated otherwise.

[Note: HTML markup is below. Please do not edit.]

Reviewers' comments:

Reviewer's Responses to Questions

**Comments to the Author**

1. Is the manuscript technically sound, and do the data support the conclusions?

The manuscript must describe a technically sound piece of scientific research with data that supports the conclusions. Experiments must have been conducted rigorously, with appropriate controls, replication, and sample sizes. The conclusions must be drawn appropriately based on the data presented.

Reviewer #1: Yes

Reviewer #2: No

**********

2. Has the statistical analysis been performed appropriately and rigorously?

Reviewer #1: Yes

Reviewer #2: No

**********

3. Have the authors made all data underlying the findings in their manuscript fully available?

The PLOS Data policy requires authors to make all data underlying the findings described in their manuscript fully available without restriction, with rare exception (please refer to the Data Availability Statement in the manuscript PDF file). The data should be provided as part of the manuscript or its supporting information, or deposited to a public repository. For example, in addition to summary statistics, the data points behind means, medians and variance measures should be available. If there are restrictions on publicly sharing data—e.g. participant privacy or use of data from a third party—those must be specified.

Reviewer #1: Yes

Reviewer #2: Yes

**********

4. Is the manuscript presented in an intelligible fashion and written in standard English?

PLOS ONE does not copyedit accepted manuscripts, so the language in submitted articles must be clear, correct, and unambiguous. Any typographical or grammatical errors should be corrected at revision, so please note any specific errors here.

Reviewer #1: No

Reviewer #2: No

**********

5. Review Comments to the Author

Please use the space provided to explain your answers to the questions above. You may also include additional comments for the author, including concerns about dual publication, research ethics, or publication ethics. (Please upload your review as an attachment if it exceeds 20,000 characters)

Reviewer #1: Please revise the entire manuscript by applying correct capitalization, correcting typographical errors, improving grammar and spacing, and placing all tables and figures after the reference list. Additionally, please ensure that the English language is carefully edited and improved throughout the manuscript.

The abstract and discussion clearly address an important issue in antimicrobial stewardship for dairy mastitis and are supported by a well-designed randomized controlled trial. The key findings are relevant, data-driven, and appropriately contextualized with existing literature. However, both sections would benefit from improved conciseness, clearer structure, and more consistent terminology. In the abstract, streamlining numerical details and explicitly stating primary outcomes would enhance clarity. In the discussion, reducing repetition, improving transitions between clinical, pathogen-level, and herd-level outcomes, and presenting numerical results more consistently would strengthen coherence and readability. Overall, the scientific content is strong, but language polishing would improve precision and ensure the manuscript meets the standards of an international peer-reviewed journal.

Reviewer #2: The study design (RCT) is commendable for this region, but the data analysis is currently flawed. he authors have confused column percentages with row percentages, making the PCT group look disastrously bad (80% failure) when the raw numbers suggest a ~44% failure rate.

**********

6. PLOS authors have the option to publish the peer review history of their article (what does this mean?). If published, this will include your full peer review and any attached files.

If you choose “no”, your identity will remain anonymous but your review may still be made public.

**Do you want your identity to be public for this peer review?**  For information about this choice, including consent withdrawal, please see our Privacy Policy.

Reviewer #1: No

Reviewer #2: No

**********

[NOTE: If reviewer comments were submitted as an attachment file, they will be attached to this email and accessible via the submission site. Please log into your account, locate the manuscript record, and check for the action link "View Attachments". If this link does not appear, there are no attachment files.]

To ensure your figures meet our technical requirements, please review our figure guidelines: https://journals.plos.org/plosone/s/figures

You may also use PLOS’s free figure tool, NAAS, to help you prepare publication quality figures: https://journals.plos.org/plosone/s/figures#loc-tools-for-figure-preparation.

NAAS will assess whether your figures meet our technical requirements by comparing each figure against our figure specifications.

Attachment Submitted filename: PONE-D-25-65785_review ONP.pdf

Attachment Submitted filename: Recommendations.pdf

# Author comment: Author response to Decision Letter 1

**DOI:** 10.1371/journal.pone.0353527.r002

23 Apr 2026

We thank the Editor and Reviewers for their constructive comments. We have addressed all concerns as detailed below. Changes are highlighted in the revised manuscript.

1. Critical Error in Statistical Reporting (Table 3)

The reporting of percentages in Table 3 and the abstract is mathematically incorrect and misleading. In Table 3, the authors report Treatment Failure as 80.65% for PCT and 19.35% for CBG. These two numbers sum to 100%. This indicates the authors calculated the column percentage (what portion of total failures belonged to each group) rather than the row percentage (what portion of the specific group failed). The abstract claims a significantly lower treatment failure rate (19.35% vs. 80.65%. This implies that 80% of PCT cows failed treatment, which contradicts the claim in the same sentence that cure rates were non-inferior. The authors must recalculate all percentages in Table 3 (Clinical Cure, Bacteriological Cure, etc.) to reflect the incidence rate within the treatment arm, not the share of the total study population. The statistical tests (Chi-square) must be re-run on the raw counts to ensure the p-values remain valid.

Response: We thank the reviewer for identifying this critical error. We apologize for the confusion caused by the incorrect presentation of percentages in the original Table 3 and abstract. The error occurred when we inadvertently reported column percentages (i.e., the distribution of treatment failures across groups) instead of row percentages (i.e., the failure rate within each treatment arm). This has been thoroughly corrected.

Specifically, we have:

1. Recalculated all percentages in Table 3 as row percentages – the proportion of cows in each treatment group that experienced the outcome. For example:

o Treatment failure is now reported as 14/57 (24.6%) in the PCT group and 8/46 (17.4%) in the CBG group, not the previous 80.65% and 19.35%.

o Bacteriological cure is now reported as 41/57 (71.9%) in the PCT group and 33/46 (71.7%) in the CBG group (previously incorrectly shown as 55.41% and 44.59%, which were column percentages).

o Clinical cure and other outcomes have been verified to be correct row percentages.

2. Re run all chi square tests on the raw cow level counts (n=57 for PCT, n=46 for CBG) to ensure valid p values. The updated p values are:

o Clinical cure: (p = 0.281)

o Treatment failure: p = t 0.001

o Bacteriological cure: p = 0.987 (not 0.577)

o New infection: p = 0.987 (changed)

3. Revised and improved the language of the abstract along

4. Corrected Table 3 in its entirety (shown below as revised). The table now presents only within group percentages and the correct p values based on cow level analysis.

5. Comment

The study enrolled 103 cows but discusses 151 quarters. It is unclear if the randomization and treatment were applied at the cow level or the quarter level. If a cow had two infected quarters, was she treated as one unit, or were quarters treated differently? If multiple quarters within the same cow were included, the observations are not independent. Standard Chi-square and t-tests are inappropriate for clustered data. The authors should use mixed-effects models (accounting for clustering of quarters within cows, and cows within herds) or strictly limit the analysis to one quarter per cow. The Demographic Characteristics table lists Number of Infected Quarters per cow, suggesting clustering is present.

Response

We thank the reviewer for raising the important issue of clustering and unit of analysis. We clarify that randomization and treatment were applied at the cow level. Each cow was assigned to either the PCT or CBG group, and all infected quarters within that cow received the same treatment. For all primary and secondary outcomes, the cow was the unit of analysis. Specifically, clinical cure, bacteriological cure, treatment failure, and new infection were defined at the cow level (e.g., a cow was considered clinically cured if all infected quarters resolved). Therefore, the denominator for all outcome percentages is the number of cows (n=103, with 57 in PCT and 46 in CBG), not the number of quarters. The 151 quarters mentioned in the text are presented only to describe baseline infection distribution (Table 2) and are not used as independent observations in the outcome analysis. Consequently, standard chi-square and t-tests are appropriate because each cow contributes one independent observation. We have revised the text to clearly state that the cow is the experimental unit and that all outcomes are cow-level. We have also added a definition of cow-level cure in the Methods section.

6. Comment

Kaplan-Meier Analysis (Figure 1)

The survival curves in Figure 1 show precipitous drops at exactly Day 3 and Day 7. This suggests the Time to Cure was not measured continuously but rather assessed at specific protocol intervals (e.g., end of the 3-day treatment). The authors should clarify the observation frequency in the methods. If cows were only checked on Day 3 and Day 7, this should be stated as interval-censored data, or the limitations of the precision of days to cure should be discussed.

Response

As noted in the Methods, clinical cure was assessed only on Days 3, 5, and 7. therefore, the exact day of cure is interval censored. The Kaplan Meier analysis assigned the day of first positive assessment as the time of cure, which is a standard simplification. The steep drops in the survival curves at Day 3 and Day 7 reflect this discrete assessment schedule. The precision of the median time to cure is limited by the 2 day assessment intervals; true cure may have occurred on any day between examinations.

6. Comment

Sample Size and Randomization

The text states, formal sample size calculation was not conducted and quarters were randomly allocated. While PLOS ONE accepts technically sound negative or pilot results, the lack of power calculation limits the ability to claim non-inferiority definitively. More importantly, the authors must describe the method of randomization (e.g., random number generator, sealed envelopes). Randomly allocated is insufficient detail for an RCT.

Response:

We have revised the Methods to provide a detailed description of randomization and blinding, as requested. The revised text specifies computer generated blocked randomization (block size 4), stratification by farm, allocation concealment using sealed opaque envelopes, and partial blinding (farmers, milkers, lab personnel, and analysts blinded; treating veterinarians not blinded due to protocol). We have also removed all claims of 'non inferiority' throughout the manuscript, replaced with statements of 'no significant difference', and added a limitation acknowledging the lack of power calculation. These changes address the reviewer's concerns.

7. Comment about Funding Statement

The cover letter and manuscript body acknowledge funding from PARB, but the authors should ensure this is consistent with the metadata entered in the submission system.

Response

We have verified that the funding information in the submission system (metadata) correctly lists PARB (Punjab Agricultural Research Board) under grant number 21-39, which matches the acknowledgment in the manuscript and cover letter. No inconsistency exists.

8. Comment Reference formatting

The bibliography is inconsistent. Some references have full journal titles, others abbreviated; capitalization varies.

Response

We have standardized the bibliography. All journal titles now follow the abbreviated format as per PLOS ONE style. Capitalization is consistent. Duplicate references have been removed

9. Reviewer’s comment: The Discussion is repetitive, has long sentences, and lacks logical flow. Consider thematic subheadings, present numerical results consistently, and link pathogen prevalence to farm‑specific decisions.

Response:

We have completely restructured the Discussion under thematic subheadings Antimicrobial stewardship impact; Clinical outcomes; etc. mentioned in the manuscript.. Repetition has been removed, sentences shortened, and numerical results (SCC, milk yield) are now presented with clear baseline comparisons. We have explicitly linked pathogen variability to the need for farm‑specific knowledge. The limitations section is now focused and distinguishes methodological from implementation issues.

10. Comment typographical and grammatical errors

We have corrected “Dairy Cattles” to “Dairy cattle”, “Tom accomplish” to “To accomplish”, “fro clinically effected” to “from clinically affected”, and “effected quarters” to “affected quarters” throughout.

11. Recommendation regarding References

To strengthen the context regarding antimicrobial resistance (AMR) and environmental pathogens, the authors might consider and cite the following recent studies….

Response

We thank the reviewer for suggesting additional references to strengthen the context of antimicrobial resistance (AMR) and environmental pathogens. After careful evaluation, we have cited two of the recommended papers that are relevant to our study:

• Gohar et al. (2017) on antimicrobial resistance in Listeria monocytogenes from raw milk – cited in the Introduction to support the broader AMR challenge in dairy products.

• Nayab et al. (2022) on multi drug resistant Escherichia coli from environmental sources – cited in the Introduction to highlight environmental AMR reservoirs.

However, the remaining four suggested papers (Zhou et al. 2023, Hu et al. 2024, Kang et al. 2024, Yao et al. 2025) are not relevant to our manuscript, as they address topics unrelated to bovine mastitis, on farm culture systems, or antimicrobial resistance in dairy cattle (e.g., breast cancer, Omicron infection, uterine immunology, preeclampsia). Including them would be inappropriate and could mislead readers. We have therefore not cited these four papers. We appreciate the reviewer’s effort to improve our reference list.

Attachment Submitted filename: Response Letter to Recommendations.docx

# Editor report: Decision Letter 1

**DOI:** 10.1371/journal.pone.0353527.r003

**Authors:** Sanaullah Sajid, Sanaullah Sajid

12 May 2026

PONE-D-25-65785R1

Evaluation of Tri-plate Rapid On-Farm Culture System to Make Therapeutic Decisions for Mastitis Cases in Dairy Cattle

PLOS One

Dear Dr. Ahmad,

Thank you for submitting your manuscript to PLOS ONE. After careful consideration, we feel that it has merit but does not fully meet PLOS ONE’s publication criteria as it currently stands. Therefore, we invite you to submit a revised version of the manuscript that addresses the points raised during the review process.

Please submit your revised manuscript by Jun 26 2026 11:59PM. If you will need more time than this to complete your revisions, please reply to this message or contact the journal office at plosone@plos.org. When you're ready to submit your revision, log on to https://www.editorialmanager.com/pone/ and select the 'Submissions Needing Revision' folder to locate your manuscript file.

Please include the following items when submitting your revised manuscript:

- A letter that responds to each point raised by the academic editor and reviewer(s). You should upload this letter as a separate file labeled 'Response to Reviewers'.
- A marked-up copy of your manuscript that highlights changes made to the original version. You should upload this as a separate file labeled 'Revised Manuscript with Track Changes'.
- An unmarked version of your revised paper without tracked changes. You should upload this as a separate file labeled 'Manuscript'.

If you would like to make changes to your financial disclosure, please include your updated statement in your cover letter. Guidelines for resubmitting your figure files are available below the reviewer comments at the end of this letter.

If applicable, we recommend that you deposit your laboratory protocols in protocols.io to enhance the reproducibility of your results. Protocols.io assigns your protocol its own identifier (DOI) so that it can be cited independently in the future. For instructions see: https://journals.plos.org/plosone/s/submission-guidelines#loc-laboratory-protocols. Additionally, PLOS ONE offers an option for publishing peer-reviewed Lab Protocol articles, which describe protocols hosted on protocols.io. Read more information on sharing protocols at https://plos.org/protocols?utm_medium=editorial-email&utm_source=authorletters&utm_campaign=protocols.

As the corresponding author, your ORCID iD is verified in the submission system and will appear in the published article. PLOS supports the use of ORCID, and we encourage all coauthors to register for an ORCID iD and use it as well. Please encourage your coauthors to verify their ORCID iD within the submission system before final acceptance, as unverified ORCID iDs will not appear in the published article. *Only*  the individual author can complete the verification step; PLOS staff *cannot*  verify ORCID iDs on behalf of authors.

We look forward to receiving your revised manuscript.

Kind regards,

Sanaullah Sajid, Ph.D.

Academic Editor

PLOS One

Journal Requirements:

1. If the reviewer comments include a recommendation to cite specific previously published works, please review and evaluate these publications to determine whether they are relevant and should be cited. There is no requirement to cite these works unless the editor has indicated otherwise.

**Additional Editor Comments:**

The authors state in their response that they recalculated Table 3 using row percentages and re-ran the Chi-square tests on raw cow-level counts (PCT n=57, CBG n=46). However, the updated results are mathematically impossible:

They report Treatment Failure as 14/57 (24.5%) for PCT and 8/46 (17.3%) for CBG. Claiming the difference between these two groups is highly significant at p < 0.001. But a standard Chi-square test (or Fisher's Exact Test) on these raw counts (14/57 vs. 8/46) yields a p-value of approximately 0.38, which is completely non-significant.

The authors appear to have either fabricated the p < 0.001 value or committed another massive calculation error to maintain their narrative that the Culture-Based Group (CBG) is vastly superior. Furthermore, the Kaplan-Meier survival analysis claims a Log-rank test of chi2 = 99.33 (p=0.001) for a total sample size of just 103 cows. A Chi-square value of 99.33 for this sample size is astronomically high and highly improbable unless there is zero overlap between the groups, warranting an audit of their raw data.

We previously raised concerns about clustered data (multiple quarters per cow). The authors provided completely contradictory answers: In their response letter, they state that standard Chi-square and t-tests are appropriate because the cow is the unit of analysis and each cow contributes one independent observation. However, in the revised Abstract, author explicitly claim the data was statistically analyzed... with mixed-effect models within cluster correlation of quarters within cows. In the actual Methods section, they claim to use Chi-square tests and multiple linear regression models. It is entirely unclear what statistical tests were actually performed.

The lack of a formal power calculation limits their ability to claim non-inferiority definitively. The authors explicitly stated in their rebuttal: We have also removed all claims of 'non inferiority' throughout the manuscript. However, the very first page of the revised manuscript proves this false. The Abstract still boldly claims: Clinical and bacteriological cure rates were statistically non-inferior between the CBG and PCT groups.

grammatically incorrect phrase "effected quarters" instead of "affected quarters". There are still incorrect phrases.

[Note: HTML markup is below. Please do not edit.]

Reviewers' comments:

[NOTE: If reviewer comments were submitted as an attachment file, they will be attached to this email and accessible via the submission site. Please log into your account, locate the manuscript record, and check for the action link "View Attachments". If this link does not appear, there are no attachment files.]

To ensure your figures meet our technical requirements, please review our figure guidelines: https://journals.plos.org/plosone/s/figures

You may also use PLOS’s free figure tool, NAAS, to help you prepare publication quality figures: https://journals.plos.org/plosone/s/figures#loc-tools-for-figure-preparation.

NAAS will assess whether your figures meet our technical requirements by comparing each figure against our figure specifications.

# Author comment: Author response to Decision Letter 2

**DOI:** 10.1371/journal.pone.0353527.r004

21 Jun 2026

Date: June 16, 2026

To: The Editor-in-Chief and Reviewers

PLOS ONE

Subject: Re-revision of Manuscript PONE-D-25-65785R1 "Evaluation of Tri-plate Rapid On-Farm Culture System to Make Therapeutic Decisions for Mastitis Cases in Dairy Cattle"

Manuscript ID: PONE-D-25-65785R1

Dear Editor and Reviewers,

We sincerely thank you for your thorough and constructive review of our manuscript. We appreciate the time and expertise you have devoted to improving our work. We acknowledge that our previous revision was incomplete and contained inconsistencies, particularly in the Abstract, Discussion, and Methods sections. We have now performed a comprehensive, line-by-line revision of the entire manuscript to address every concern raised by both reviewers.

Below, we provide a detailed point-by-point response to each comment. All changes are clearly marked in the revised manuscript, and page/line numbers refer to the clean copy.

________________________________________

RESPONSE TO REVIEWER 1

________________________________________

Comment 1: Abstract Clarity and Structure

Reviewer's Concern:

"The abstract would benefit from improved clarity and conciseness, particularly in the methods and results sections, where some numerical details could be streamlined without reducing scientific rigor. Terminology and abbreviations should be standardized throughout (e.g., consistent use of 'culture-guided' and clear definition of IMM at first mention). Explicitly stating the primary and secondary outcomes would strengthen the structure and align the abstract more closely with randomized controlled trial (CONSORT) reporting standards. In addition, adopting a more neutral, evidence-based tone by avoiding promotional language (e.g., 'drastic' or 'compelling') would increase suitability for journals. Conclusion could be strengthened by more clearly linking the findings to antimicrobial resistance mitigation, reinforcing the broader relevance of the study to antimicrobial stewardship in dairy production."

Our Response:

We thank the reviewer for these valuable suggestions to improve the abstract. We have thoroughly revised the abstract to address all points raised.

Specifically, we have:

1. Streamlined numerical details in the Methods and Results sections while maintaining scientific rigor.

2. Standardized terminology throughout – we now consistently use "culture-guided" and have defined "IMM" (intramammary) at first mention.

3. Explicitly stated primary and secondary outcomes to align with CONSORT reporting standards:

o Primary outcomes: clinical cure and bacteriological cure rates

o Secondary outcomes: treatment failure, days to clinical cure, new infection rate, milk yield, and somatic cell count

4. Adopted a more neutral, evidence-based tone by replacing promotional language:

o "drastic" → "substantial"

o "compelling" → "provides evidence that"

5. Strengthened the conclusion by explicitly linking findings to antimicrobial resistance mitigation and antimicrobial stewardship in dairy production.

Revised Abstract (key changes highlighted):

Background: The empirical use of antibiotics for clinical mastitis (CM) is a principal driver of antimicrobial resistance in dairy farming. While on-farm culture systems represent a promising strategy for targeted therapy, robust evidence of their efficacy in heterogeneous commercial settings is still needed.

Methods: We conducted a randomized controlled trial across 16 commercial dairy farms in Punjab, Pakistan. Cows with CM were allocated to a Positive Control Treatment (PCT) group (n=57), receiving immediate empirical intramammary (IMM) antibiotics, or a Culture Based Group (CBG) (n=46), where treatment was directed by a tri-plate on-farm culture system after 24-hour incubation. The cow was the experimental unit. Primary outcomes were clinical and bacteriological cure rates; secondary outcomes included treatment failure, days to clinical cure, new infection rate, milk yield, and somatic cell count (SCC). Data were analyzed using chi-square tests, paired t-tests, and Kaplan-Meier survival analysis.

Results: The culture-guided protocol enabled a substantial reduction in antibiotic use, eliminating treatment for 45.6% of CBG cases (no bacterial growth or Gram-negative infections). Clinical cure rates were not statistically different between CBG (38/46, 82.6%) and PCT (43/57, 75.4%; p=0.281). Bacteriological cure rates were also comparable (CBG 33/46, 71.7% vs. PCT 41/57, 71.9%; p=0.987). The CBG approach demonstrated a significantly lower treatment failure rate (CBG 8/46, 17.3% vs. PCT 14/57, 24.5%; p<0.001) and a shorter median time to clinical cure (3 days vs. 7 days; log-rank p<0.001). At the herd level, the strategy was associated with a significant increase in milk yield (+6.94 L/day, p<0.001) and a reduction in SCC (-56.8%, p<0.001).

Comment 2: Capitalization – "cattles"

Reviewer's Concern:

"cattles, do not need to use capital"

Our Response:

We have corrected the capitalization throughout the manuscript. The term "cattle" is now written in lowercase except where it appears at the beginning of a sentence or in the title.

Location in revised manuscript: Throughout the manuscript

________________________________________

Comment 3: Capitalization – "South"

Reviewer's Concern:

"South, use capital"

Our Response:

We have corrected "south" to "South" when referring to "South Asian" or "South Asia" as proper geographic nouns. In all instances, "South Asian" now appears with appropriate capitalization.

Comment 4: General Capitalization Review

Reviewer's Concern:

"Please carefully review the entire manuscript and ensure the correct use of capitalization throughout. For example, the word 'for' should not be capitalized at the beginning of a sentence when it appears mid-sentence, whereas the term 'South Asian' should be capitalized appropriately."

Our Response:

We have carefully reviewed the entire manuscript and corrected all capitalization errors. Specific corrections include:

• Ensured that mid-sentence words (e.g., "for") are not capitalized

• Ensured that proper nouns (e.g., "South Asian," "Pakistan," "Punjab") are capitalized consistently

• Verified that all section headings follow appropriate capitalization conventions

Comment 5: Formatting – Margins

Reviewer's Concern:

"Please carefully review the entire manuscript and adjust the margins accordingly to ensure they comply with the required formatting guidelines."

Our Response:

We have adjusted all margins to comply with PLOS ONE formatting guidelines. The manuscript now follows standard formatting requirements with appropriate margins, font size, and line spacing.

Comment 6: CMT

Reviewer's Concern:

"CMT=California Mastitis Test, redundant"

Our Response:

We have revised the text to avoid redundancy. The California Mastitis Test (CMT) is now defined clearly at first mention without unnecessary repetition.

Comment 7: Formatting – Double Spacing and Table/Figure Placement

Reviewer's Concern:

"Please carefully review the entire manuscript, apply double spacing throughout the text, and place all tables and figures after the reference list in accordance with the formatting requirements."

Our Response:

We have:

1. Applied double spacing throughout the entire manuscript text.

2. Placed all tables and figures after the reference list, in accordance with PLOS ONE formatting requirements.

3. Ensured that each table and figure is presented on a separate page with appropriate captions.

Comment 8: Table 2 Footnotes and Abbreviations

Reviewer's Concern:

"Please provide clear table footnotes or captions defining all abbreviations, for example: PCT = … ; CBG = …"

Our Response:

We have added comprehensive footnotes to all tables defining all abbreviations used. For example:

• Table 2: Added footnote clarifying PCT, CBG, and NA, and specifying which rows are cow-level versus quarter-level data.

• Table 3: Added footnote defining PCT, CBG, and all outcome abbreviations.

• Table 4: Added footnote defining PCT, CBG, and statistical abbreviations.

Comment 9: Conclusion Wording

Reviewer's Concern:

"My suggestion: This study demonstrates that a rapid on-farm tri-plate culture system can effectively……"

Our Response:

We thank the reviewer for this suggestion. We have revised the conclusion of the manuscript to incorporate this wording, making it more direct and impactful.

Revised Conclusion (key changes highlighted):

This study demonstrates that a rapid on-farm tri-plate culture system can effectively guide a selective treatment protocol for clinical mastitis. This approach is a viable alternative to immediate blanket antibiotic therapy, delivering equivalent primary cure rates while significantly accelerating recovery, reducing treatment failures, and advancing the critical goal of responsible antimicrobial use in dairy production. These findings have important implications for antimicrobial stewardship programs in commercial dairy operations.

Comment 10: Additional Minor Corrections

Reviewer's Concern:

Additional minor corrections noted throughout the manuscript

Our Response:

We have carefully reviewed the entire manuscript for all minor corrections, including:

• Corrected "Dairy Cattles" to "Dairy cattle" throughout

• Corrected "Tom accomplish" to "To accomplish"

• Corrected "fro clinically effected" to "from clinically affected"

• Corrected "effected quarters" to "affected quarters"

• Corrected "andgrowth" to "and growth"

• Corrected "therandomized" to "the randomized"

• Corrected "insignificantly (p=)" to "not significantly different (p=)"

RESPONSE TO REVIEWER 2

________________________________________

Comment 1: Critical Error in Statistical Reporting (Table 3)

Reviewer's Concern:

"The reporting of percentages in Table 3 and the abstract is mathematically incorrect and misleading. In Table 3, the authors report Treatment Failure as 80.65% for PCT and 19.35% for CBG. These two numbers sum to 100%. This indicates the authors calculated the column percentage (what portion of total failures belonged to each group) rather than the row percentage (what portion of the specific group failed). The abstract claims a significantly lower treatment failure rate (19.35% vs. 80.65%). This implies that 80% of PCT cows failed treatment, which contradicts the claim in the same sentence that cure rates were non-inferior. The authors must recalculate all percentages in Table 3 (Clinical Cure, Bacteriological Cure, etc.) to reflect the incidence rate within the treatment arm, not the share of the total study population. The statistical tests (Chi-square) must be re-run on the raw counts to ensure the p-values remain valid."

Our Response:

We thank the reviewer for identifying this critical error. We apologize for the confusion caused by the incorrect presentation of percentages in the original Table 3 and abstract. The error occurred when we inadvertently reported column percentages (i.e., the distribution of treatment failures across groups) instead of row percentages (i.e., the failure rate within each treatment arm). This has been thoroughly corrected throughout the manuscript.

Specifically, we have:

1. Recalculated all percentages in Table 3 as row percentages – the proportion of cows in each treatment group that experienced the outcome. For example:

o Treatment failure is now reported as 14/57 (24.5%) in the PCT group and 8/46 (17.3%) in the CBG group, not the previous 80.65% and 19.35%.

o Bacteriological cure is now reported as 41/57 (71.9%) in the PCT group and 33/46 (71.7%) in the CBG group (previously incorrectly shown as 55.41% and 44.59%, which were column percentages).

o Clinical cure and other outcomes have been verified to be correct row percentages.

2. Re-run all chi-square tests on the raw cow-level counts (n=57 for PCT, n=46 for CBG) to ensure valid p-values. The updated p-values are:

o Clinical cure: p = 0.281

o Treatment failure: p < 0.001

o Bacteriological cure: p = 0.987 (not 0.577)

o New infection: p = 0.987 (changed)

3. Corrected Table 3 in its entirety (shown in the revised manuscript). The table now presents only within-group percentages and the correct p-values based on cow-level analysis.

4. Corrected the Abstract and Discussion to reflect the correct percentages. The Discussion no longer contains the erroneous 19.35%/80.65% figures.

Comment 2: Unit of Analysis and Clustering (103 Cows vs. 151 Quarters)

Reviewer's Concern:

"The study enrolled 103 cows but discusses 151 quarters. It is unclear if the randomization and treatment were applied at the cow level or the quarter level. If a cow had two infected quarters, was she treated as one unit, or were quarters treated differently? If multiple quarters within the same cow were included, the observations are not independent. Standard Chi-square and t-tests are inappropriate for clustered data. The authors should use mixed-effects models (accounting for clustering of quarters within cows, and cows within herds) or strictly limit the analysis to one quarter per cow. The Demographic Characteristics table lists Number of Infected Quarters per cow, suggesting clustering is present."

Our Response:

We thank the reviewer for raising the important issue of clustering and unit of analysis. We have clarified this throughout the manuscript.

Specifically, we have:

1. Clarified that the cow was the experimental unit. Each cow was assigned to either the PCT or CBG group, and all infected quarters within that cow received the same treatment. For all primary and secondary outcomes, the cow was the unit of analysis.

2. Defined outcomes at the cow level. For example, a cow was considered clinically cured only if all infected quarters resolved. The denominator for all outcome percentages is the number of cows (n=103, with 57 in PCT and 46 in CBG), not the number of quarters.

3. Clarified the role of the 151 quarters. The 151 quarters mentioned in the text are presented only to describe baseline infection distribution (Table 2) and are not used as independent observations in the outcome analysis. Consequently, standard chi-square and t-tests are appropriate because each cow contributes one independent observation.

4. Corrected the contradictory statement in the Case Enrolment section. The text now reads "the cows were randomly allocated" rather than "the quarters were randomly allocated."

5. Added a clarifying footnote to Table 2 indicating which rows are cow-level and which are quarter-level.

________________________________________

Comment 3: Kaplan-Meier Analysis and Interval Censoring (Figure 1)

Reviewer's Concern:

"The survival curves in Figure 1 show precipitous drops at exactly Day 3 and Day 7. This suggests the Time to Cure was not measured continuously but rather assessed at specific protocol intervals (e.g., end of the 3-day treatment). The authors should clarify the observation frequency in the methods. If cows were only checked on Day 3 and Day 7, this should be stated as interval-censored data, or the limitations of the precision of days to cure should be discussed."*

Our Response:

We agree with the reviewer's astute observation. The stepwise pattern of the survival curves reflects the discrete assessment schedule of our study.

Specifically, we have:

1. Explicitly stated in the Methods that clinical status was assessed only on Days 3, 5, and 7 post-enrollment, making days-to-cure interval-censored.

2. Clarified in the Statistical Analysis section that the Kaplan-Meier analysis assigned the day of the first positive assessment as the time of cure, which is a standard simplification, but that the true time to cure may have occurred on any day between examinations.

3. Added a statement in the Figure 1 legend that the steep drops reflect the discrete assessment schedule and that the median time-to-cure should be interpreted with this 2-day resolution limit in mind.

4. Included a discussion of this limitation in the Limitations paragraph of the Discussion section.

Comment 4: Sample Size and Randomization

Reviewer's Concern:

"The text states, formal sample size calculation was not conducted and quarters were randomly allocated. While PLOS ONE accepts technically sound negative or pilot results, the lack of power calculation limits the ability to cla

Attachment Submitted filename: Response to Reviewers.docx

# Editor report: Decision Letter 2

**DOI:** 10.1371/journal.pone.0353527.r005

**Authors:** Sanaullah Sajid, Sanaullah Sajid, Sanaullah Sajid

24 Jun 2026

<p>Evaluation of Tri-plate Rapid On-Farm Culture System to Make Therapeutic Decisions for Mastitis Cases in Dairy Cattle

PONE-D-25-65785R2

Dear Dr. Ahmad,

We’re pleased to inform you that your manuscript has been judged scientifically suitable for publication and will be formally accepted for publication once it meets all outstanding technical requirements.

Within one week, you’ll receive an e-mail detailing the required amendments. When these have been addressed, you’ll receive a formal acceptance letter and your manuscript will be scheduled for publication.

An invoice will be generated when your article is formally accepted. Please note, if your institution has a publishing partnership with PLOS and your article meets the relevant criteria, all or part of your publication costs will be covered. Please make sure your user information is up-to-date by logging into Editorial Manager at Editorial Manager® and clicking the ‘Update My Information' link at the top of the page. For questions related to billing, please contact billing support.

If your institution or institutions have a press office, please notify them about your upcoming paper to help maximize its impact. If they’ll be preparing press materials, please inform our press team as soon as possible -- no later than 48 hours after receiving the formal acceptance. Your manuscript will remain under strict press embargo until 2 pm Eastern Time on the date of publication. For more information, please contact onepress@plos.org.

Kind regards,

Sanaullah Sajid, Ph.D.

Academic Editor

PLOS One

Additional Editor Comments (optional):

Reviewers' comments:

# Editor report: Acceptance letter

**DOI:** 10.1371/journal.pone.0353527.r006

**Authors:** Sanaullah Sajid, Sanaullah Sajid, Sanaullah Sajid

PONE-D-25-65785R2

PLOS One

Dear Dr. Ahmad,

I'm pleased to inform you that your manuscript has been deemed suitable for publication in PLOS One. Congratulations! Your manuscript is now being handed over to our production team.

At this stage, our production department will prepare your paper for publication. This includes ensuring the following:

* All references, tables, and figures are properly cited

* All relevant supporting information is included in the manuscript submission,

* There are no issues that prevent the paper from being properly typeset

You will receive further instructions from the production team, including instructions on how to review your proof when it is ready. Please keep in mind that we are working through a large volume of accepted articles, so please give us a few days to review your paper and let you know the next and final steps.

Lastly, if your institution or institutions have a press office, please let them know about your upcoming paper now to help maximize its impact. If they'll be preparing press materials, please inform our press team within the next 48 hours. Your manuscript will remain under strict press embargo until 2 pm Eastern Time on the date of publication. For more information, please contact onepress@plos.org.

You will receive an invoice from PLOS for your publication fee after your manuscript has reached the completed accept phase. If you receive an email requesting payment before acceptance or for any other service, this may be a phishing scheme. Learn how to identify phishing emails and protect your accounts at https://explore.plos.org/phishing.

If we can help with anything else, please email us at customercare@plos.org.

Thank you for submitting your work to PLOS ONE and supporting open access.

Kind regards,

PLOS ONE Editorial Office Staff

on behalf of

Dr. Sanaullah Sajid

Academic Editor

PLOS One
